# Factors Associated with Death during Tuberculosis Treatment of Patients Co-Infected with HIV at the Yaoundé Central Hospital, Cameroon: An 8-Year Hospital-Based Retrospective Cohort Study (2006–2013)

**DOI:** 10.1371/journal.pone.0115211

**Published:** 2014-12-15

**Authors:** Ako A. Agbor, Jean Joel R. Bigna, Serges Clotaire Billong, Mathurin Cyrille Tejiokem, Gabriel L. Ekali, Claudia S. Plottel, Jean Jacques N. Noubiap, Hortence Abessolo, Roselyne Toby, Sinata Koulla-Shiro

**Affiliations:** 1 Faculty of Medicine and Biomedical Sciences, University of Yaoundé 1, P.O. Box 1364, Yaoundé, Cameroon; 2 Goulfey Health District Unit, Ministry of Public Health, P.O. Box 62 Kousséri, Goulfey, Cameroon; 3 National AIDS control committee, Ministry of Public Health, P.O. Box 1459, Yaoundé, Cameroon; 4 Department of Epidemiology and Public Health, Centre Pasteur of Cameroun, P.O. Box 1264 Yaoundé, Cameroon, Member International Network of the Pasteur Institute; 5 Department of Medicine, New York University Langone Medical Center, New York, New York, United States of America; 6 Department of Medicine, New York University School of Medicine, New York, New York, United States of America; 7 Internal Medicine Unit, Edéa Regional Hospital, P.O. Box 100 Edéa, Cameroon; 8 Infectious Diseases Unit, Yaoundé Central Hospital, P.O. Box 5555 Yaoundé, Cameroon; FIOCRUZ, Brazil

## Abstract

**Background:**

Contributors to fatal outcomes in TB/HIV co-infected patients actively undergoing TB treatment are poorly characterized. The aim was to assess factors associated with death in TB/HIV co-infected patients during the initial 6 months of TB treatment.

**Methods:**

We conducted a hospital-based retrospective cohort study from January 2006 to December 2013 at the Yaoundé Central Hospital, Cameroon. We reviewed medical records to identify hospitalized co-infected TB/HIV patients aged 15 years and older. Death was defined as any death occurring during TB treatment, as per the World Health Organization's recommendations. We conducted logistic regression analysis to identify factors associated with a fatal outcome. Magnitudes of associations were expressed by adjusted odds ratio (a*OR*) with 95% confidence interval.

**Results:**

The 337 patients enrolled had a mean age of 39.3 (standard deviation 10.3) years and 54.3% were female. TB treatment outcomes were distributed as follows: 205 (60.8%) treatment success, 99 (29.4%) deaths, 18 (5.3%) not evaluated, 14 (4.2%) lost to follow-up, and 1 (0.3%) failed. After exclusion of patients lost to follow-up and not evaluated, death in TB/HIV co-infected patients during TB treatment was associated with a TB diagnosis made before 2010 (a*OR* = 2.50 [1.31–4.78]; *p* = 0.006), the presence of other AIDS-defining diseases (a*OR* = 2.73 [1.27–5.86]; *p* = 0.010), non-AIDS comorbidities (a*OR* = 3.35 [1.37–8.21]; *p* = 0.008), not receiving cotrimoxazole prophylaxis (a*OR* = 3.61 [1.71–7.63]; *p* = 0.001), not receiving antiretroviral therapy (a*OR* = 2.45 [1.18–5.08]; *p* = 0.016), and CD4 cells count <50 cells/mm^3^ (a*OR* = 16.43 [1.05–258.04]; *p* = 0.047).

**Conclusions:**

The TB treatment success rate among TB/HIV co-infected patients in our setting is low. Mortality was high among TB/HIV co-infected patients during TB treatment and is strongly associated with clinical and biological factors, highlighting the urgent need for specific interventions focused on enhancing patient outcomes.

## Introduction

Tuberculosis (TB) due to *Mycobacterium tuberculosis* and human immunodeficiency virus (HIV) disease have been inextricably linked from the earliest years of the HIV/acquired immunodeficiency syndrome (AIDS) epidemic [1], [2]. Their dangerous synergy affects all aspects of both diseases, from pathogenesis and the epidemiologic profile, to clinical presentation, treatment, and prevention. This synergy also impacts larger societal issues with demographic, economic, and even political consequences [3].

The prevalence of HIV in Cameroon was estimated at 4.5% in 2012 [4]. In 2012, the number of HIV/AIDS related deaths reached 35,000 [4] with TB being the leading cause of death [5]–[7]. Based on an autopsy series in Botswana, 40 to 65% of HIV-infected patients with respiratory disease also harbored TB [8]. According to the World Health Organization (WHO), Cameroon which has a TB incidence rate of 124 cases per 100,000 inhabitants [9], is categorized as an intermediate TB burden country [10]. In 2013, 38% of TB patients in the country were infected with HIV [9].

In Cameroon, the treatment success rate in TB/HIV co-infected patients stands at 65% [9]. Despite improved treatment success rates, the death rate from TB in HIV co-infected persons remains very high. While some studies have found lower TB cure rates (58.8%) among TB/HIV patients compared to patients infected with TB alone (78.8%) [11], [12], other studies reported similar TB cure rates in those TB/HIV co-infected as compared to those infected with TB alone [13], [14]. Death rates in TB patients from sub-Saharan countries where HIV is highly prevalent have risen substantially over the last few decades [6]. A significant proportion of these deaths occur early in the course of treatment [7], [15]–[18] threatening the credibility of public health interventions to control TB in the eyes of the patients, health care providers, and the community. We therefore sought to evaluate the outcomes of TB treatment in TB/HIV co-infected patients with a focus on the factors associated with death during treatment.

Using a cohort design, we conducted an eight-year (2006–2013) hospital-based retrospective study at the Infectious Disease Unit (IDU) of the Yaoundé Central Hospital (YCH) to determine socio-demographical, clinical, and biological factors associated with death among TB/HIV co-infected patients undergoing TB treatment. We anticipate that data will contribute in improving the management of TB/HIV patients in Cameroon.

## Materials and Methods

### Design and setting

We conducted a hospital-based retrospective cohort study at the Infectious Disease Unit (IDU) of the Yaoundé Central Hospital (YCH) in Cameroon. The YCH is a tertiary-care hospital, which serves as a university teaching hospital for both undergraduate and postgraduate medical education. It offers specialized care for patients with infectious diseases along with various other pathologies, and has the greatest pool of HIV-infected patients in Yaoundé and its surrounding area. The IDU serves as the referral unit for the management of adults diagnosed with TB infection at the YCH. There are 238 Centers for Diagnosis and Treatment (CDT) of TB in Cameroon. The IDU houses one of the 22 TB CDTs in Yaoundé and thus treats and manages 10% of all TB cases diagnosed in the city, corresponding to 2% of all TB cases in Cameroon, per year [19]. All diagnosed TB cases are started on the standard TB treatment used in Cameroon [19]; outcomes are reported according to national TB control guidelines [19]. Patient medical information from initial consultation to discharge is recorded in a registry, the Tuberculosis Reporting Registry, which is the official system for mandatory reporting of all hospital TB cases.

### Population

TB cases were identified via the Tuberculosis Reporting Register of the IDU of the YCH, the official system for mandatory reporting of all hospital TB cases. At diagnosis, TB reporting is mandatory nationwide in Cameroon for new cases or retreatment cases [19]. All patients reported from January 1, 2006 to June 30, 2013 were selected for inclusion in the study. This period was chosen in order to yield the maximum amount of data based on availability of the archives at the IDU of the YCH. Charts and data were located, retrieved, confirmed, and any identified duplicative files were consolidated. We included all TB/HIV co-infected patients who had undergone tuberculosis treatment with or without ART. The duration of follow-up for each patient was 6 months from initiation of the antituberculous therapeutic regimen, corresponding to the entire duration of TB treatment for new cases, and to the first 6 months of the 8-month regimen for retreated cases.

**Figure 1:**
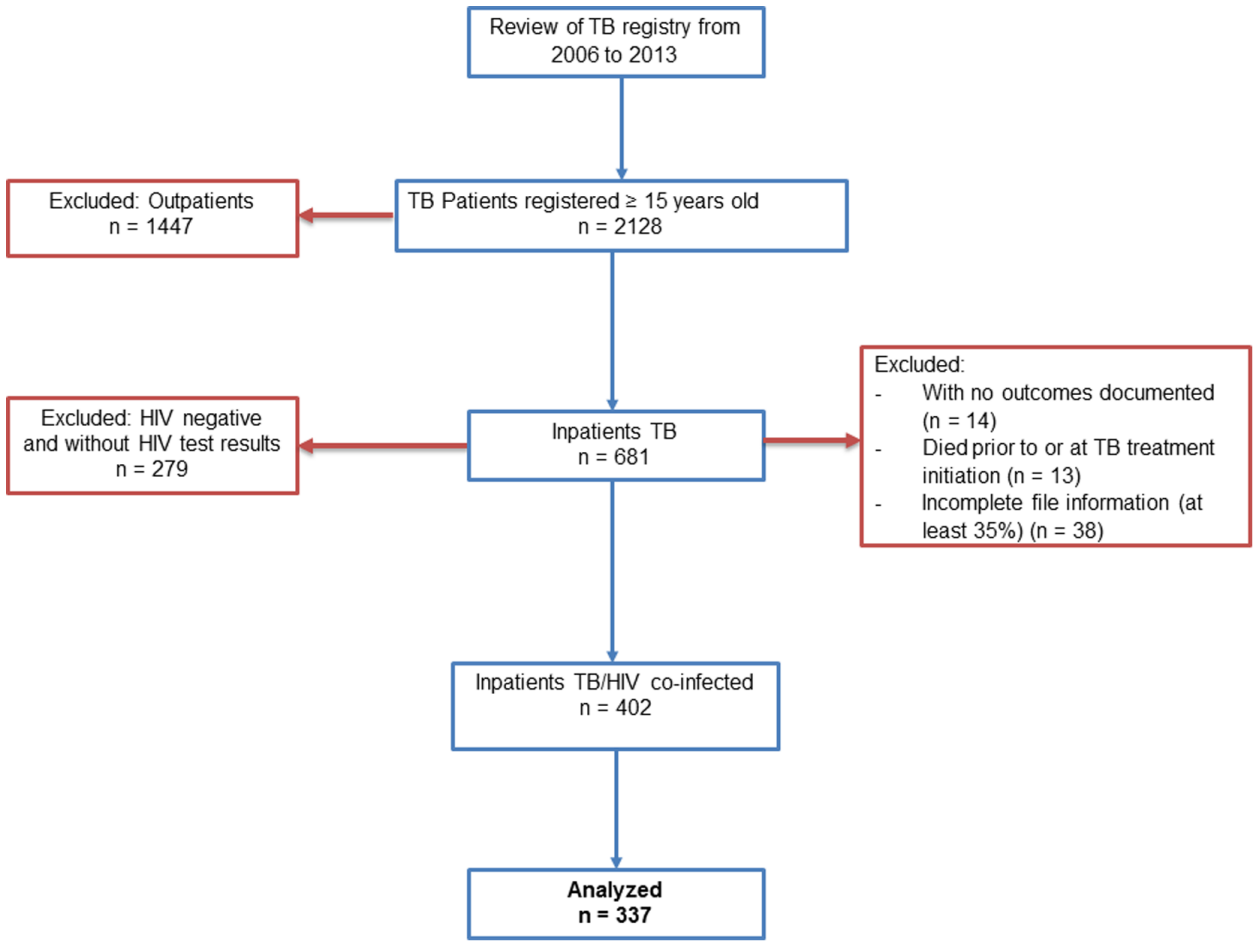
Enrollment of study participants.

We screened 2,128 records from the TB Reporting Register, as schematized in Fig. 1, and included HIV-infected patients with TB, aged 15 years and older. We excluded outpatients, patients who died before initiation of TB treatment, or on the date TB treatment began, cases with missing information on follow-up and outcomes in the IDU/TB registers, and patients with incomplete file information (at least 35% of variables). Our study included only hospitalized patients because most of the outpatients fell under our exclusion criteria due to incomplete information in greater than 70%. After exclusions, 337 patients remained eligible for analysis and constituted our cohort of TB/HIV co-infected hospitalized subjects.

### Data collection

#### Procedure

Data were extracted from: (i) the TB Reporting Register of the IDU-YCH, (ii) hospitalization registry of the IDU, (iii) medical records of hospitalized TB/HIV co-infected patients and (iv) TB yellow cards of TB/HIV co-infected patients. A TB yellow card is a physical record (on A4 format paper) used in Cameroon for documenting ongoing tuberculosis treatment and that is retained at health center and handled by caregivers [19]. It contains demographic and administrative information (name, sex, age, address, TB registry number) along with the type of TB (pulmonary versus extra-pulmonary) at the start of treatment, HIV status, and a calendar with check boxes for each day of taking anti-TB medication.

#### Socio-demographic data

We reviewed the patients' medical records and extracted socio-demographic data, including age, sex, marital status, highest level of education, and residence.

#### Clinical data

We collected data on the timing of TB and HIV diagnoses. The physician-in-charge of the CDT classified each patient's clinical TB presentation at the time of diagnosis according to national guidelines [19] adapted from the WHO Standard International Definitions [10]: *smear-positive pulmonary TB* (Acid-fast bacilli [AFB] originally found in at least two sputum specimens), *smear-negative pulmonary TB* (persistence of negative result of three sputum examinations after ten days of non-specific antibiotic treatment in a patient with clinical and radiographic signs suggesting pulmonary TB without other obvious cause), *and extra-pulmonary TB* (a patient in whom TB was found in organs other than the lungs [e.g. pleura, lymph nodes, abdomen, genitourinary tract, skin, joints and bones, meninges]). We considered as mixed form patients those who had extra-pulmonary disease associated with any form of pulmonary TB. At the IDU, most of the diagnoses of extra-pulmonary TB were based on clinical assessment (because of limited access to specific diagnostic tools) and on a favorable response to empirical anti-TB treatment.

National guidelines [19] adopted from the WHO Standard International Definitions [10] were used to classify TB status at the time of diagnosis. Each TB cases was classified as a *new case* or a *retreatment case*. Other clinical variables retrieved from patients' medical records included body weight, the occurrence of other AIDS-related opportunistic diseases, co-morbidities that were neither TB nor AIDS-related diseases, information on the different treatment outcomes, and whether or not the patient received Cotrimoxazole Prophylactic Therapy (CPT) and ART.

#### Biological data

We also reviewed patients' laboratory results and retrieved total white blood cell and CD4 cell counts, and hemoglobin level.

#### TB treatment outcomes

At the IDU of the YCH, all patients receiving TB treatment are reassessed at the end of months 2, 5 and 6 for new cases, and at the end of months 3, 5 and 8 in cases of retreatment. Thus, all information on treatment outcome was obtained from patients' medical records and their TB yellow cards. Outcomes were classified by the physician in-charge of the CDT using the definitions from the TB program in Cameroon [19] adapted from WHO international standard definitions [10] as follow: *Cured* (an initially sputum smear-positive patient whose sputum was smear-negative in the last month of treatment and on at least one previous occasion), *Completed Treatment* (a TB patient who completed treatment without evidence of failure but with no documentation that sputum smear or culture results in the last month of treatment and on at least one previous occasion were negative, either because tests were not done or because the results were unavailable), *Died* (from any cause during treatment), *Failed* (a patient whose sputum was initially smear-positive and remained sputum smear-positive at month 5 or later during TB treatment), *Lost to follow-up* (A patient who did not start treatment or whose treatment was interrupted for two consecutive months or more), and *Not evaluated* (a TB patient for whom no treatment outcome was assigned. This includes cases ‘transferred out’ to another CDT of TB as well as cases for whom the treatment outcome is unknown to the reporting unit). *Successfully treated* was a patient who was cured or who completed treatment.

### Ethics statement

We obtained an ethical clearance from the Institutional Review Board of the Faculty of Medicine and Biomedical Sciences, University of Yaoundé 1, Cameroon. An authorization of the YCH was also obtained before data collection. The data were collected and analyzed anonymously.

### Data analysis

Data was extracted, coded, entered, and analyzed using the Statistical Package for Social Science (SPSS) version 20.0 for Windows (IBM Corp. Released 2011. IBM SPSS Statistics for Windows, Version 20.0. Armonk, NY: IBM Corp.). Continuous variables were expressed as the mean with standard deviation (SD) or median with interquartile range (IQR). Categorical variables were expressed as frequency with percentages (%). Frequency of TB treatment outcomes was analyzed using Windows Program for Epidemiologists version 11.25 and reported with Fisher's exact 95% confidence interval (95%CI).

To analyze predictors of death, we excluded in the first analysis, all patients who were *lost to follow-up* and *not evaluated* because we had no information on the outcome (death or survival). We also did a secondary analysis on attrition, excluding only *not evaluated* patients and considering *lost to follow-up* as *death*. In a third analysis, all continuous variables have not been categorized. Then, multiple imputation was used to handle missing data, creating a new data set which was the average of five data sets of imputed values [20], [21]. We included in the imputation model only variables assessed as risk factors of death. We therefore did not calculate imputed data for treatment outcome and we excluded those patients from all analysis as it was our primary outcome. We classified any death that occurred during TB treatment as a TB death, consistent with WHO guidelines [10]. We conducted univariate analysis comparing death and non-death patients concerning socio-demographic, clinical and biological characteristics. After this, we performed binary multivariate regression analyses to identify independent predictors of death. The initial multivariate logistic regression model included non colinear variables associated with death (as dependent variable) if the p-value was ≤0.20 in the univariate analysis. The final model was obtained by successively removing variables not associated at a p-value <0.05 only if the odds ratios for remaining variables were unchanged and taking interactions into account. Known risk factors such as weight, CD4 count, ART, or sampling variables such as year of inclusion were maintained systematically in the final model.

## Results

### Description of study population

Of the 681 TB inpatients, 402 (59.0%) were HIV co-infected and 279 (41.0%) were free of HIV infection or had negative HIV test results (Fig. 1). Of the 337 eligible patients, 180 (53.4%) were female. Mean age was 39.3 years (SD 10.3). Most of patients (n = 149, 44.2%) had a secondary education. There were mostly married patients (n = 155, 46.0%). Most patients resided in an urban area (n = 301, 89.3%). When considering the clinical TB presentation, most patients had extra-pulmonary TB alone (n = 125, 37.1%), followed closely by cases of smear-positive pulmonary TB alone (n = 119, 35.3%). The majority of our cases represented new diagnoses of tuberculosis (n = 297, 88.1%). Four fifths of patients received CPT and around two thirds received ART (Table 1).

**Table 1:** Socio-demographic, clinical, and biological characteristics of 337 patients co-infected with TB and HIV, Yaoundé Central Hospital, 2006–2013, Cameroon.

Variables	
**SOCIO-DEMOGRAPHIC**	
Sex	
Male	46.6% (n = 157)
Female	53.4% (n = 180)
Age	
Range	18–78 years
Mean	39.3 years (10.3)
Median	38 years (32–46)
Educational level	
No formal education	14.2% (n = 48)
Primary	14.2% (n = 48)
Secondary	44.2% (n = 149)
University	27.3% (n = 92)
Residence	
Rural	10.7% (n = 36)
Urban	89.3% (n = 301)
Marital status	
Married/Cohabitating	46.0% (n = 155)
Single	44.8% (n = 151)
Widowed	6.8% (n = 23)
Divorced	2.4% (n = 8)
**CLINICAL**	
TB presentation*	
SPP TB only	35.3% (n = 119)
SNP TB only	22.3% (n = 75)
EP TB only	37.1% (n = 125)
Mixed (Pulmonary + EP TB)	5.3% (n = 18)
Status at TB diagnosis	
New case	88.1% (n = 297)
Relapse	7.7% (n = 26)
Re-treatment after failure	2.1% (n = 7)
Defaulter	0.3% (n = 1)
Other	1.8% (n = 6)
Weight^1^	
Mean	53.2 Kg (10.3)
Median	53 Kg (45–60)
Known duration of HIV infection	
Mean	8.4 Months (19.4)
Median	0.95 Months (0.13–5.7)
Other AIDS-related non-TB disease	15.7% (n = 50)
Other non-AIDS co-morbidity	11.9% (n = 40)
Received prophylactic cotrimoxazole	79.5% (n = 268)
Received antiretroviral therapy	67.4% (n = 227)
**LABORATORY VALUES**	
White blood cell count^2^	
Mean	6471 cells/mm^3^ (5210)
Median	5100 cells/mm^3^ (3300–7990)
Hemoglobin^3^	
Mean	8.3 g/dl (2.4)
Median	8 g/dl (7–10)
CD4 cell count^4^	
Mean	121 cells/mm^3^ (109)
Median	102 cells/mm^3^ (33–178)

Data are % (n), mean (standard deviation) or median (interquartile range).

TB: tuberculosis.

*SPP: smear positive pulmonary, SNP: smear negative pulmonary, EP: extra pulmonary.

1 Data missing: there were 36 (10.7%) records without recorded weights.

2 Data missing: there were 18 (5.3%) records without recorded white blood cell counts.

3 Data missing: there were 18 (5.3%) records without recorded hemoglobin values.

4 Data missing: there were 28 (8.3%) records without recorded CD4 cell counts.

### TB treatment outcomes

**Figure 2:**
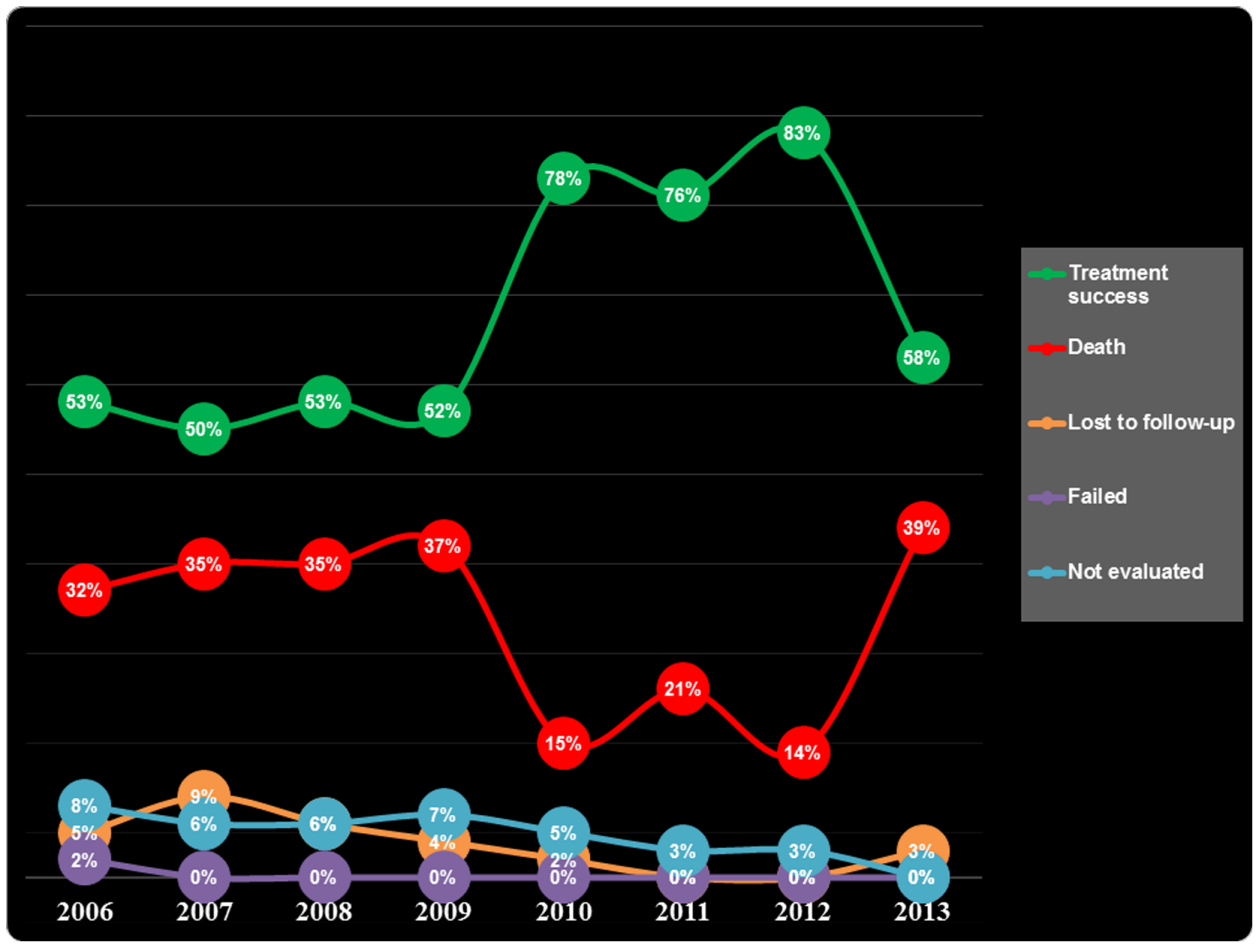
Trend of TB treatment outcomes by year in TB/HIV co-infected patients from 2006 to 2013.

**Figure 3:**
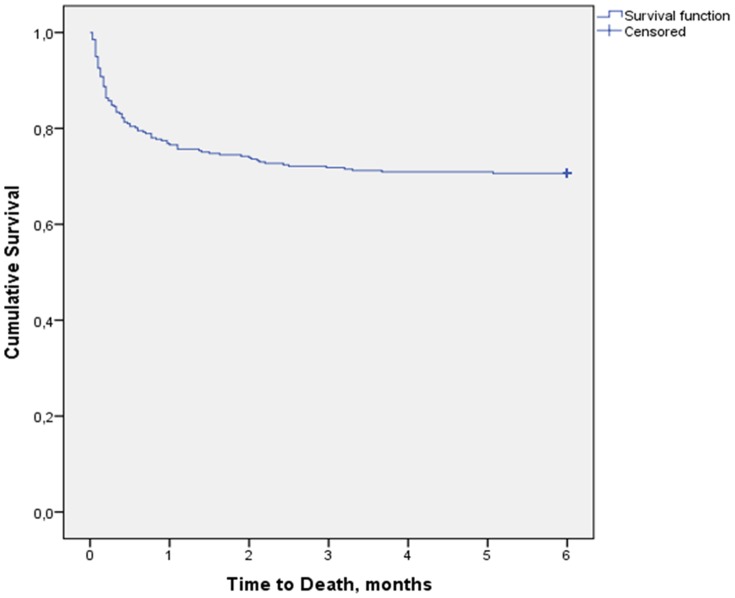
Kaplan-Meier survival curve of TB/HIV co-infected patients on TB treatment (N = 337).

Among the 337 patients, TB treatment outcomes were distributed as follow: 134 (39.8%, 95%CI 34.5–45.2) *completed treatment*, 71 (21.1%, 95%CI 16.9–25.9) were *cured*, 99 (29.4%, 95%CI 24.6–34.6) *died*, 18 (5.3%, 95%CI 3.3–8.5) were *not evaluated*, 14 (4.2%, 95%CI 2.4–7.0) *lost to follow-up* and 1 (0.3%, 95%CI 0.02–1.9) *failed*. The overall *treatment success* rate (completed treatment or cured) was 60.8% (95% CI 55.4–66.0). Fig. 2 presents the trend of TB treatment outcomes in TB/HIV co-infected patients over the period from 2006 to 2013. In this figure, the trend of treatment success (green line) is symmetric to the trend of death (red line). After exclusion of *not evaluated* and *lost to follow-up* patients, among the remaining 305 patients who were followed for 6 months, the death rate was 32.5% (95%CI 27.2–38.0). Fig. 3 presents the Kaplan-Meier survival curve of the overall TB/HIV co-infected patients. We observed that after the 2^nd^ month of TB treatment, the survival curve remains flat till the end of the 6^th^ month. Death rates at one month, at two months, and at five months were 23.4%, 26.1% and 29.4% respectively.

### Factors associated with death during TB treatment

In our first method of analysis (Table 2), in univariate analysis, factors associated with death during TB treatment were: TB diagnosis between 2006 and 2009, having body weight <50 Kgs, having a duration of known HIV infection ≥12 months, the presence of an AIDS-defining disease other than TB, the presence of an non-AIDS related comorbidity, not receiving CPT, not receiving ART, and having a CD4 cell count <50/mm^3^. In multivariate analysis, factors that remained associated with death during TB treatment were TB diagnosis between 2006 and 2009 (a*OR*: 2.50, 95%CI 1.31–4.78; *p* = 0.006), the presence of another AIDS-defining disease besides TB (a*OR*: 2.73, 95%CI 1.27–5.86; *p* = 0.010), the presence of another non-AIDS defining illness (a*OR*: 4.04, 95%CI 1.37–8.21; *p* = 0.008), not receiving CPT (a*OR*: 3.61, 95%CI 1.71–7.63; *p* = 0.001), not receiving ART (a*OR*: 2.45, 95%CI 1.18–5.08; *p* = 0.016), and having CD4 cell count <50/mm^3^ (a*OR*: 16.43, 95%CI 1.05–258.04; *p* = 0.047).

**Table 2:** Factors associated with death during TB treatment among TB/HIV co-infected patients, Yaoundé Central Hospital, 2006–2013, Cameroon.

Variables, n (%)	Dead n = 99	Alive n = 206	Total N = 305§	Univariate analysis		Multivariate analysis	
				Crude OR (CI 95%)	p-value	Adjusted OR (CI 95%)	p-value
**SOCIO-DEMOGRAPHIC**							
Sex							
Male	52 (52.5)	96 (46.6)	148 (48.5)	1.27 (0.78–2.05)	.333		
Female	47 (47.5)	110 (53.4)	157 (51.5)	Ref			
Age (years)							
≥47	26 (26.3)	47 (22.8)	73 (23.9)	1.22 (0.62–2.39)	.567		
39–46	20 (20.2)	58 (28.2)	78 (25.6)	0.76 (0.38–1.52)	.435		
33–38	28 (28.3)	46 (22.3)	74 (23.3)	1.34 (0.69–2.61)	.390		
18–32	25 (25.3)	55 (26.7)	80 (26.2)	Ref			
Level of education							
Primary/No formal	30 (30.3)	56 (27.2)	86 (28.2)	1.16 (0.69–1.97)	.571		
Secondary/University	69 (69.7)	150 (72.8)	219 (71.8)	Ref			
Marital status							
Alone (single/widowed/divorced)	60 (60.6)	103 (50.0)	163 (53.4)	1.54 (0.95–2.50)	.083	1.44 (0.78–2.65)	.242
Married/Cohabiting	39 (39.4)	103 (50.0)	142 (46.6)	Ref			
Residence							
Rural	13 (13.1)	21 (10.2)	34 (11.1)	0.75 (0.40–1.57)	.447		
Urban	86 (86.9)	185 (89.8)	271 (88.9)	Ref			
**CLINICAL**							
Year of TB diagnosis							
2006–2009	70 (70.7)	106 (51.5)	176 (57.7)	2.28 (1.37–3.80)	.002	2.50 (1.31–4.78)	.006
2010–2013	29 (29.3)	100 (48.5)	129 (42.3)	Ref			
TB clinical presentation*							
Mixed (Pulmonary + EP TB)	8 (8.1)	7 (3.4)	15 (4.9)	2.91 (1.86–4.56)	.973		
EP TB only	41 (41.4)	69 (33.5)	110 (36.1)	1.51 (1.20–1.91)	.859		
SNP TB only	19 (19.2)	51 (24.8)	70 (23.0)	0.95 (0.72–1.25)	.485		
SPP TB only	31 (31.3)	79 (38.3)	110 (36.1)	Ref			
Status at TB diagnosis							
Retreatment case	11 (11.1)	27 (13.1)	38 (12.5)	0.83 (0.61–1.12)	.227		
New case	88 (88.9)	179 (86.9)	267 (87.5)	Ref			
Body weight (Kg)							
≤50	51 (51.5)	63 (30.6)	114 (37.4)	2.38 (1.44–3.93)	.001	1.75 (0.94–3.29)	.079
>50	39 (39.4)	117 (56.8)	156 (51.1)	Ref			
* Missing*	9 (9.1)	26 (12.6)	35 (11.5)	-			
Duration of known HIV infection							
≥12 months	13 (13.1)	39 (18.9)	52 (17.0)	0.65 (0.49–0.85)	.002	0.73 (0.32–1.68)	.460
<12 months	86 (86.9)	167 (81.1)	253 (83.0)	Ref			
Presence of another AIDS-related non-TB disease							
Yes	27 (27.3)	23 (11.2)	50 (16.4)	2.98 (2.32–3.84)	<.0001	2.73 (1.27–5.86)	.010
No	72 (72.7)	183 (88.8)	255 (83.6)	Ref			
Presence of another non-AIDS comorbidity							
Yes	20 (20.2)	17 (8.3)	37 (12.1)	2.81 (2.12–3.74)	<.0001	3.35 (1.37–8.21)	.008
No	79 (79.8)	189 (91.7)	268 (87.9)	Ref			
Cotrimoxazole prophylactic therapy							
No	32 (32.3)	31 (15.0)	63 (20.7)	2.70 (2.14–3.40)	<.0001	3.61 (1.71–7.63)	.001
Yes	67 (67.7)	175 (85.0)	242 (79.0)	Ref			
Antiretroviral therapy							
No	37 (37.4)	60 (29.1)	97 (31.8)	1.45 (1.18–1.79)	.0004	2.45 (1.18–5.08)	.016
Yes	62 (62.6)	146 (70.9)	208 (68.2)	Ref			
**LABORATORY VALUES**							
White blood cell level (cell/mm^3^)							
<4,000	38 (38.4)	61 (29.6)	99 (32.5)	1.67 (0.98–2.84)	.061	1.32 (0.68–2.58)	.417
>10,000	14 (14.1)	26 (12.6)	40 (13.1)	1.39 (0.67–2.87)	.375	0.54 (0.20–1.46)	.220
4,000–10,000	40 (40.4)	110 (53.4)	150 (49.2)	Ref			
* Missing*	7 (7.1)	9 (4.4)	16 (5.2)	-			
Hemoglobin level (g/dl)							
<8	39 (39.4)	61 (29.6)	100 (32.8)	1.55 (0.91–2.64)	.107	1.33 (0.70–2.54)	.380
≥8	53 (53.5)	135 (65.5)	188 (61.6)	Ref			
* Missing*	7 (7.1)	10 (4.9)	17 (5.6)	-			
CD4 cell count (cell/mm^3^)							
<50	56 (56.6)	44 (21.4)	100 (32.8)	8.14 (0.75–88.89)	.082	16.43 (1.05–258.04)	.047
50–199	31 (31.3)	100 (48.5)	131 (43.0)	2.09 (0.20–21.71)	.519	4.17 (0.29–59.8)	.275
200–350	4 (4.0)	33 (16.0)	37 (12.1)	1.09 (0.06–19.28)	.950	1.40 (0.06–31.52)	.821
>350	1 (1.0)	10 (4.9)	11 (3.6)	Ref			
* Missing*	7 (7.1)	19 (9.2)	26 (8.5)	-			

§ From the 337 patients, we have excluded all patients who were *lost to follow-up* (n = 14) and *not evaluated* (n = 18).

*SPP: smear positive pulmonary, SNP: smear negative pulmonary, EP: extra pulmonary.

TB: tuberculosis.

All missing data were imputed.

In the analysis on attrition (Table 3), in univariate analysis, factors associated with death/lost to follow-up (LTFU) during TB treatment were: being single, a TB diagnosis between 2006 and 2009, having mixed form of TB at clinical presentation, having body weight <50 Kgs, the presence of an AIDS-defining disease other than TB, the presence of an non-AIDS related comorbidity, and not receiving CPT. In multivariate analysis, factors associated with death/LTFU during TB treatment were TB diagnosis between 2006 and 2009 (a*OR*: 3.0, 95%CI 1.57–5.70; *p* = 0.001), the presence of another AIDS-defining disease besides TB (a*OR*: 2.61, 95%CI 1.20–5.70; *p* = 0.016), the presence of another non-AIDS defining illness (a*OR*: 4.04, 95%CI 1.37–8.21; *p* = 0.008), not receiving CPT (a*OR*: 3.91, 95%CI 1.86–8.21; *p*<0.001), not receiving ART (a*OR*: 2.64, 95%CI 1.29–5.38; *p* = 0.008), and having CD4 cell count <50/mm^3^ (a*OR*: 11.14, 95%CI 1.82–68.21; *p* = 0.011).

**Table 3:** Factors associated with death/lost to follow-up during TB treatment among TB/HIV co-infected patients, Yaoundé Central Hospital, 2006–2013, Cameroon.

Variables, n (%)	Dead+LTFUn = 113	Alive n = 206	Total N = 319§	Univariate analysis		Multivariate analysis	
				Crude OR (CI 95%)	p-value	Adjusted OR (CI 95%)	p-value
**SOCIO-DEMOGRAPHIC**							
Sex							
Male	54 (47.8)	96 (46.6)	150 (53.0)	0.95 (0.60–1.51)	.839		
Female	59 (52.2)	110 (53.4)	169 (47.0)	Ref			
Age (years)							
≥47	26 (23.0)	47 (22.8)	73 (22.9)	1.05 (0.54–2.02)	.886		
39–46	25 (22.1)	58 (28.2)	83 (26.0)	0.82 (0.43–1.57)	.543		
33–38	33 (29.2)	46 (22.3)	79 (24.8)	1.36 (0.72–2.57)	.341		
18–32	29 (25.7)	55 (26.7)	84 (26.3)	Ref			
Level of education							
Primary/No formal	35 (31.0)	56 (27.2)	91 (28.5)	1.20 (0.72–1.99)	.474		
Secondary/University	78 (69.0)	150 (72.8)	228 (71.5)	Ref			
Marital status							
Alone (single/widowed/divorced)	70 (69.1)	103 (50.0)	173 (54.2)	1.63 (1.02–2.60)	.041	1.67 (0.91–3.04)	.097
Married/Cohabiting	43 (38.1)	103 (50.0)	146 (45.8)	Ref			
Residence							
Rural	13 (11.5)	21 (89.8)	34 (10.7)	1.15 (0.55–2.38)	.717		
Urban	100 (88.5)	185 (10.2)	285 (89.3)	Ref			
**CLINICAL**							
Year of TB diagnosis							
2006–2009	82 (72.6)	106 (51.5)	188 (58.9)	2.50 (1.52–4.10)	<.001	3.0 (1.57–5.70)	.001
2010–2013	31 (27.4)	100 (48.5)	131 (41.1)	Ref			
TB clinical presentation*							
Mixed (Pulmonary + EP TB)	10 (8.8)	7 (3.4)	17 (5.3)	2.89 (1.02–8.18)	.045	1.74 (0.47–6.48)	.407
EP TB only	43 (38.1)	69 (33.5)	112 (35.1)	1.26 (0.74–2.17)	.398	1.49 (0.76–2.91)	.241
SNP TB only	21 (18.6)	51 (24.8)	72 (22.6)	0.83 (0.44–1.58)	.577	0.48 (0.21–1.06)	.069
SPP TB only	39 (34.5)	79 (38.3)	118 (37.0)	Ref			
Status at TB diagnosis							
Retreatment case	12 (10.6)	27 (13.1)	39 (12.2)	0.79 (0.38–1.62)	.517		
New case	101 (89.4)	179 (86.9)	280 (87.8)	Ref			
Body weight (Kg)							
≤50	56 (49.6)	63 (30.6)	119 (37.3)	2.11 (1.27–3.50)	.004	1.46 (0.79–2.69)	.226
>50	47 (41.6)	117 (56.8)	164 (51.4)	Ref			
* Missing*	10 (8.8)	26 (12.6)	36 (11.3)	-			
Duration of known HIV infection							
≥12 months	14 (12.4)	39 (18.9)	53 (16.6)	1.65 (0.85–3.19)	.136	1.31 (0.59–2.90)	.513
<12 months	99 (87.6)	167 (81.1)	266 (83.4)	Ref			
Presence of another AIDS-related non-TB disease							
Yes	29 (25.7)	23 (11.2)	52 (16.3)	2.74 (1.50–5.03)	.001	2.61 (1.20–5.70)	.016
No	84 (74.3)	183 (88.8)	267 (83.7)	Ref			
Presence of another non-AIDS comorbidity							
Yes	21 (18.6)	17 (8.3)	38 (11.9)	2.54 (1.28–5.04)	.008	3.34 (1.40–7.99)	.007
No	92 (81.4)	189 (91.7)	281 (88.1)	Ref			
Cotrimoxazole prophylactic therapy							
No	36 (31.9)	31 (15.0)	67 (21.0)	2.64 (1.52–4.57)	.001	3.91 (1.86–8.21)	<.001
Yes	77 (68.1)	175 (85.0)	252 (79.0)	Ref			
Antiretroviral therapy							
No	43 (38.1)	60 (29.1)	103 (32.3)	1.50 (0.92–2.43)	.104	2.64 (1.29–5.38)	.008
Yes	70 (61.9)	146 (70.9)	216 (67.7)	Ref			
**LABORATORY VALUES**							
White blood cell level (cell/mm^3^)							
<4,000	41 (36.3)	61 (29.6)	102 (32.0)	1.49 (0.88–2.51)	.135	1.26 (0.65–2.43)	.499
>10,000	17 (15.0)	26 (12.6)	43 (13.5)	1.57 (0.78–3.17)	.206	0.71 (0.29–1.76)	.453
4,000–10,000	47 (41.6)	110 (53.4)	157 (49.2)	Ref			
* Missing*	8 (7.1)	9 (4.4)	17 (5.3)	-			
Hemoglobin level (g/dl)							
<8	42 (37.2)	61 (29.6)	103 (32.3)	1.45 (0.90–2.35)	.129	1.28 (0.70–2.34)	.426
≥8	63 (55.8)	136 (66.0)	199 (62.4)	Ref			
* Missing*	8 (7.1)	9 (4.4)	17 (5.3)	-			
CD4 cell count (cell/mm^3^)							
<50	62 (54.9)	44 (21.4)	106 (33.2)	3.81 (0.89–16.24)	.069	11.14 (1.82–68.21)	.011
50–199	36 (31.9)	100 (48.5)	136 (42.6)	1.03 (0.22–4.74)	.967	2.59 (0.44–15.44)	.283
200–350	4 (3.5)	33 (16.0)	37 (11.6)	0.44 (0.07–2.90)	.379	0.61 (0.09–4.13)	.604
>350	3 (2.7)	10 (4.9)	13 (4.1)	Ref			
* Missing*	8 (7.1)	19 (9.2)	27 (8.5)	-			

§ From the 337 patients, we have excluded all patients who were *not evaluated* (n = 18).

*SPP: smear positive pulmonary, SNP: smear negative pulmonary, EP: extra pulmonary.

LTFU: lost to follow-up, TB: tuberculosis.

All missing data were imputed.

In the third analysis (S1 Data), integrating age, body weight, duration of known HIV infection, white blood cell level, hemoglobin level, and CD4 cell count without categorization, factors associated with death were TB diagnosis between 2006 and 2009 (aOR: 2.55, 95%CI 0.81–2.64; p = 0.003), the presence of another AIDS-defining disease besides TB (a*OR*: 2.39, 95%CI 1.14–5.02; *p* = 0.022), the presence of another non-AIDS defining illness (a*OR*: 2.83, 95%CI 1.22–.60; *p* = 0.016), not receiving CPT (a*OR*: 3.31, 95%CI 1.61–6.80; *p* = 0.001), not receiving ART (a*OR*: 2.41, 95%CI 1.20–4.84; *p* = 0.014), and CD4 cell count (a*OR* [1 cell decrease]: 1.01, 95%CI 1.006–1.013; *p*<0.0001).

## Discussion

In this retrospective tertiary-care hospital-based study in Cameroon, the TB treatment success rate among TB/HIV co-infected patients is low (60.8%), possibly reflecting a high death rate (29.4%). However, we noted an improvement in the treatment success rates over time, from 2006 to 2013. Moreover, co-infected patients in our setting demonstrated lower rates of LTFU and failure as compared to that reported in other studies [7], [9], [17], [22]–[24]. The present study reveals several factors associated with death in TB/HIV co-infected patients ongoing TB treatment at the IDU of the YCH. They include four clinical factors: the presence of another opportunistic disease, the presence of another non-AIDS related comorbidity, not receiving CPT, and not receiving ART; and one biological factor: having a CD4 cell count <50/mm^3^. The study did not identify an association with any socio-demographic factors. A TB diagnosis between 2006 and 2009 was also a factor associated with death within 6 months of TB treatment initiation. The same factors were found when we added LTFU to death.

### Death rate

The overall death rate in our cohort of patients was 29.4% (95%CI 24.6–34.6), which is notably higher than that reported in previous studies including inpatients and outpatients in Cameroon (9.9% [95%CI 7.4–12.8] and 7.4% [95%CI 3.6–13.1]) [11], [23], in Nigeria (11.1% [95%CI 7.0–16.5]) [25], and in India (15.0%, 95%CI 14.4–15.9) [26]. Most patients requiring hospitalization for TB therapy (as is the case in our cohort) are generally sicker. They present with advanced infection and severe immune deficiency, such that in the absence of rapid intervention, death becomes imminent. Most of the deaths in our cohort occurred within the first 4 weeks after TB treatment initiation. This is congruent with findings from several studies where death in TB/HIV co-infected patients tended to occur early in the course of TB treatment [15], [16], [18], [27]. The fact that patients, especially those suspected of having extra-pulmonary TB were often unable to pay for and obtain needed diagnostic testing contributed to delays in TB diagnosis and treatment. A number of possible explanations may underlie the observed increased mortality among our co-infected patients. The location and extent of TB is influenced by the degree of host immunosuppression, often increasing the difficulty of diagnosis and hence delaying treatment initiation, resulting in higher mortality [28]. Immunological studies have also shown that host responses to *Mycobacterium tuberculosis* enhance HIV replication [29], [30], thus accelerating the natural progression of HIV and further depressing cellular immunity. From the year 2006 to 2009, the death rate in our study population was stable, remaining between 32% and 35%. It dropped to about 15% in 2010 and later rose to about 39% in 2013. Again, a great proportion of patients in the 2013 cohort were still on TB treatment and their outcomes had not yet being assigned to them at the time of data collection data for the present study. Another contributing factor was several occurrences of inadequate supplies and stock-outs of anti-tuberculosis drugs at the IDU in the year 2013.

Death rates in TB patients particularly in the high HIV prevalence populations of sub-Saharan African countries including Cameroon, have risen substantially over the last decades [6]. It is worth noting that HIV infection has been shown to be an independent predictor of death in TB patients during TB treatment [7], [31], [32] and may represent the major reason why the 29.4% overall mortality rate in our cohort of patients was as high as it was. Sileshi and colleagues [16] in Northwest Ethiopia similarly found that the risk of death during TB treatment is higher in patients treated as inpatients compared to those treated at an outpatient health center. As mentioned, a likely reason is that those who are cared for in the inpatient setting have more extensive disease. As a result, the more severely ill hospitalized patients have a greater mortality, as compared to the less ill health center outpatients. This observation also likely explains the reason for the high incidence of death in our study population treated at the IDU of the YCH. The symmetric trend of success rate and death suggest that the frequency of death rate was possibly related to success rate but not to other outcomes of TB treatment. The overall death rate in our cohort of patients is also notably higher than that reported in another major center of tuberculosis treatment in Yaoundé (Jamot hospital) among inpatients (10–12%) [7], [24]. This is possibly due to fact that Jamot hospital is a specialized center focusing on management of TB compared to IDU-YCH. Also, among patients followed for six months, death rate was higher as compared to the death rate reported with inclusion of LTFU and not evaluated patients.

### Factors associated with death

The presence of other AIDS-related opportunistic diseases, co-morbidities, and other non-AIDS related conditions was associated with mortality. The finding that opportunistic infections and other co-morbid conditions are associated with a lethal outcome during treatment is consistent with a study carried out in Malaysia [27] and in Northern India [33]. TB is known to be the most common opportunistic infection and the leading cause of death in HIV patients worldwide, especially in resource-limited settings [5], [6]. Thus, when other AIDS-related opportunistic diseases and other co-morbid conditions are added to the burden of TB and when TB complicates the course of AIDS, the immune system weakens and death becomes inevitable in spite of anti-tuberculosis treatments. We cannot rule out the ineffectiveness of CPT and patients not receiving CPT as potential underlying contributors to the risk of death among those with other AIDS-related opportunistic diseases.

Patients not receiving CPT were more likely to die than those taking CPT. In line with this finding, studies from Sub-Saharan Africa have shown that not taking CPT was significantly associated with mortality [16], [34]–[36]. In Cameroon, CPT is indicated in all cases of TB/HIV co-infection in both adults and children, irrespective of the CD4 cell count level and WHO clinical stage [19]. All the patients in our cohort were thus eligible to CPT. Mwaungulu and colleagues in Malawi [36] found that overall TB mortality rate was reduced from 37% to 29% when patients received CPT. In their study, although TB mortality rate was unchanged over 2 years in HIV-negative patients, it decreased from 43% to 24% in HIV-infected patients. The study concluded that TB/HIV patients should systematically receive CPT. In our study, however, patients who died shortly after being diagnosed with TB and HIV may not have had the chance to initiate CPT. This may have led us to overestimate the benefit of CPT.

Not receiving ART has been well documented as a risk factor for death among TB/HIV co-infected patients in several studies [16], [26], [27], [37]. Our results are in agreement with the observation, as not receiving ART was an independent predictor of death in our cohort of TB/HIV co-infected patients undergoing TB treatment. The widespread use of antiretroviral therapy beginning in 1996 has markedly improved the survival of HIV-infected patients in both developing and developed countries by reducing the number of deaths from opportunistic infections [4]. In TB/HIV co-infected patients, introduction of ART is not an easy decision because of issues related to immune reconstitution inflammatory syndrome. However, Sileshi and colleagues in Ethiopia demonstrated that HIV-associated TB patients will have better survival if highly active ART and TB treatment are started concurrently [16], a finding consistent with the Camelia study in which the death rate was lower among patients receiving ART 2 weeks after TB treatment initiation compared to those receiving ART 8 weeks later [38]. This is also in line with recommendations from the Ministry of Public Health in Cameroon [19], [39].

The present study further revealed that TB/HIV co-infected patients with low CD4 counts <50 cells/mm^3^ were more likely to die than those with CD4 cell counts >350 cells/mm^3^. This striking observation is consistent with a study in Northwest Ethiopia, which showed that TB/HIV co-infected patients with a CD4 count of <50 cells/mm^3^ had a 13% increased risk of death compared to patients with CD4 counts greater than or equal to 200 cells/mm^3^. Other studies have reported that the highest death rates occurred in TB/HIV co-infected patients with the lowest CD4 counts [7], [27], [37], [40]. HIV infection impairs cell-mediated immunity, largely through depletion of CD4 lymphocytes. The impaired immunity leads to increased number of cases of primary TB and reactivation of TB in HIV-infected people [41]. In addition, the depletion of CD4 cells can, because of the emergence of associated illness, result in interruption of TB treatment, a risk factor for the development of drug resistant TB, the treatment of which is costly and complex, especially in the setting of HIV-infection further contributing to an increasing risk of death [17].

Interestingly, we found that a diagnosis of TB in the period from 2006 to 2009 (before 2010) was significantly associated with death as compared to a diagnosis made during other periods of our study. In 2010, Cameroon introduced updated national guidelines on ART based on the WHO recommendations and guidance [39], designed to improve the quality and enhance the delivery of care. From 2006 to 2009, ART was prescribed in the presence of active TB and CD4 count ≤350 cell/mm^3^ and can be deferred once CD4 count ≥200 cell/mm^3^. Since 2010, ART has been prescribed for persons with HIV and active TB regardless of the level of CD4 cell count. It is therefore important to prescribe ART to all TB/HIV co-infected patients as soon as possible after the TB diagnosis is established.

Certain risk factors for death we identified in our cohort are identical with those in a general HIV-infected population without TB [42]–[44]. This is explained by the fact that the emergence of TB in the setting of HIV reflects HIV-related immunosuppression and TB infection can therefore be considered a stage of HIV infection.

### Limitations

Our study has some limitations. Data about other potentially confounding biomedical predictors for death in our population, such as drug resistance, serum albumin level, time from HIV diagnosis to initiation of ART, and adherence to medication were not available and were thus excluded from the analysis [38], [45]–[47]. Data related to these potential predictors were incomplete or absent in many patient records, underscoring the significant challenges of performing ‘real life’ retrospective clinical research in Sub-Saharan Africa. For example, due to patients' lack of money, some diagnostic and laboratory testing were not performed. Furthermore, our study was limited to patients hospitalized during the study period, representing about one third of all patients with tuberculosis followed at the center during that time and two thirds of all TB/HIV co-infected patients aged 15 years and above. Our results may, therefore, not be generalizable to all patients with TB/HIV, particularly those receiving their treatment on an ambulatory basis. Exclusion of patients who were transferred out may have also confounded our results slightly since patients lost to follow-up probably included individuals dying at home without their death being reported. Duration of known HIV infection is distinct from the duration of HIV infection and therefore underestimates the true duration of HIV infection. We also acknowledge that body mass index would have provided more information that weight alone, unfortunately, we did could not get the height of patients in medical records.

## Conclusions

The death rate was high in our cohort. TB diagnosis before 2010, the presence of other AIDS-related diseases and non AIDS-related co-morbid conditions, not receiving CPT, not receiving ART, and having CD4 cell counts <50 cells/mm^3^ were identified as potential factors of death during TB treatment among TB/HIV co-infected patients.

We recommend that clinicians initiate TB treatment well before CD4 cells fall below 50 cells/mm^3^; and that they prescribe CPT for all TB/HIV co-infected patients. We also call for more clinical and scientific research in the area of TB/HIV co-infection on a larger scale, including both inpatients and outpatients in resource limited settings to assess the generalizability of our findings. For health policy makers, we advocate reinforcing training of health personnel on the diagnosis and management of TB/HIV co-infection, making TB and HIV drugs and cotrimoxazole consistently available to all those requiring them, in order to avoid stock-outs, and strengthening TB and HIV collaborative treatment activities in order to generate the knowledge that is needed for better care of TB/HIV co-infected patients.

## Supporting Information

S1 Data **Factors associated with death during TB treatment among TB/HIV co-infected patients, Yaoundé Central Hospital, 2006–2013, Cameroon (sensitivity analysis integrating continuous data).**
(DOCX)

S2 Data **Data of the 337 patients included in the study.**
(PDF)
