# Supplementary material for: Factors Associated with Death during Tuberculosis Treatment of Patients Co-Infected with HIV at the Yaoundé Central Hospital, Cameroon: An 8-Year Hospital-Based Retrospective Cohort Study (2006–2013)
Source: PLoS One. 2014 Dec 15;9(12):e115211. doi: 10.1371/journal.pone.0115211 (PMC4266669; doi:10.1371/journal.pone.0115211)
Supplement: S1 Data — Factors associated with death during TB treatment among TB/HIV co-infected patients, Yaoundé Central Hospital, 2006–2013, Cameroon (sensitivity analysis integrating continuous data). (DOCX) [file pone.0115211.s001.docx]

**Data S1. Factors associated with death during TB treatment among TB/HIV co-infected patients, Yaoundé Central Hospital, 2006-2013, Cameroon (sensitivity analysis integrating continuous data)**

|  | **Dead**  **n=99** | **Alive**  **n=206** | **Total**  **N = 305**^§^ | **Univariate analysis** | | **Multivariate analysis** | |
| --- | --- | --- | --- | --- | --- | --- | --- |
|  |  |  |  | Crude OR  (CI 95%) | p-value | Adjusted OR  (CI 95%) | p-value |
| **SOCIO-DEMOGRAPHIC** |  |  |  |  |  |  |  |
| Sex |  |  |  |  |  |  |  |
| - Male | 52 (52.5) | 96 (46.6) | 148 (48.5) | 1.27 (0.78-2.05) | .333 |  |  |
| - Female | 47 (47.5) | 110 (53.4) | 157 (51.5) | Ref |  |  |  |
| Mean age, years (SD) | 39.5 (10.9) | 39.4 (10.1) | 39.4 (10.3) | 1.00 (0.99-1.02) | .945 |  |  |
| Level of education |  |  |  |  |  |  |  |
| - Primary/No formal | 30 (30.3) | 56 (27.2) | 86 (28.2) | 1.16 (0.69-1.97) | .571 |  |  |
| - Secondary/University | 69 (69.7) | 150 (72.8) | 219 (71.8) | Ref |  |  |  |
| Marital status |  |  |  |  |  |  |  |
| - Alone (single/widowed/ divorced) | 60 (60.6) | 103 (50.0) | 163 (53.4) | 1.54 (0.95-2.50) | .083 | 1.46 (0.81-2.64) | .210 |
| - Married/Cohabiting | 39 (39.4) | 103 (50.0) | 142 (46.6) | Ref |  |  |  |
| Residence |  |  |  |  |  |  |  |
| - Rural | 13 (13.1) | 21 (10.2) | 34 (11.1) | 0.75 (0.40-1.57) | .447 |  |  |
| - Urban | 86 (86.9) | 185 (89.8) | 271 (88.9) | Ref |  |  |  |
| **CLINICAL** |  |  |  |  |  |  |  |
| Year of TB diagnosis |  |  |  |  |  |  |  |
| - 2006-2009 | 70 (70.7) | 106 (51.5) | 176 (57.7) | 2.28 (1.37-3.80) | .002 | 2.55 (1.38-4.72) | .003 |
| - 2010-2013 | 29 (29.3) | 100 (48.5) | 129 (42.3) | Ref |  |  |  |
| TB clinical presentation* |  |  |  |  |  |  |  |
| - Mixed (Pulmonary + EP TB) | 8 (8.1) | 7 (3.4) | 15 (4.9) | 2.91 (1.86-4.56) | .973 |  |  |
| - EP TB only | 41 (41.4) | 69 (33.5) | 110 (36.1) | 1.51 (1.20-1.91) | .859 |  |  |
| - SNP TB only | 19 (19.2) | 51 (24.8) | 70 (23.0) | 0.95 (0.72-1.25) | .485 |  |  |
| - SPP TB only | 31 (31.3) | 79 (38.3) | 110 (36.1) | Ref |  |  |  |
| Status at TB diagnosis |  |  |  |  |  |  |  |
| - Retreatment case | 11 (11.1) | 27 (13.1) | 38 (12.5) | 0.83 (0.61-1.12) | .227 |  |  |
| - New case | 88 (88.9) | 179 (86.9) | 267 (87.5) | Ref |  |  |  |
| Mean of body weight, Kg (SD) | 50.0 (11.2)^a^ | 54.6 (9.7)^b^ | 53.1 (10.4) | 0.96 (0.94-0.99) | .003 | 0.98 (0.95-1.01) | .254 |
| Duration of known HIV infection, years (continuous) | 0.5 (1.3) | 0.8 (1.8) | 0.7 (1.6) | 0.87 (0.73-1.05) | .139 | 0.94 (0.77-1.14) | .518 |
| Presence of another AIDS-related non-TB disease |  |  |  |  |  |  |  |
| - Yes | 27 (27.3) | 23 (11.2) | 50 (16.4) | 2.98 (2.32-3.84) | <.0001 | 2.39 (1.14-5.02) | .022 |
| - No | 72 (72.7) | 183 (88.8) | 255 (83.6) | Ref |  |  |  |
| Presence of another non-AIDS comorbidity |  |  |  |  |  |  |  |
| - Yes | 20 (20.2) | 17 (8.3) | 37 (12.1) | 2.81 (2.12-3.74) | <.0001 | 2.83 (1.22-6.60) | .016 |
| - No | 79 (79.8) | 189 (91.7) | 268 (87.9) | Ref |  |  |  |
| Cotrimoxazole prophylactic therapy |  |  |  |  |  |  |  |
| - No | 32 (32.3) | 31 (15.0) | 63 (20.7) | 2.70 (2.14-3.40) | <.0001 | 3.31 (1.61-6.80) | .001 |
| - Yes | 67 (67.7) | 175 (85.0) | 242 (79.0) | Ref |  |  |  |
| Antiretroviral therapy |  |  |  |  |  |  |  |
| - No | 37 (37.4) | 60 (29.1) | 97 (31.8) | 1.45 (1.18-1.79) | .0004 | 2.41 (1.20-4.84) | .014 |
| - Yes | 62 (62.6) | 146 (70.9) | 208 (68.2) | Ref |  |  |  |
| **LABORATORY VALUES** |  |  |  |  |  |  |  |
| Mean of white blood cell level^,^ cell/mm^3^ (SD) | 6,815.9 (7,551.7)^c^ | 6,132.2 (3,866.7)^d^ | 6,349.8 (5,319.5) | 1.00 (1.00-1.00) | .289 |  |  |
| Mean of hemoglobin level, g/dl (SD) | 7.8 (2.5)^c^ | 8.6 (2.2)^d^ | 8.3 (2.3) | 0.88 (0.79-0.98) | .019 | 0.93 (0.82-1.06) | .296 |
| Mean of CD4 cell count, cell/mm^3^ (SD) | 70.9 (82.7)^c^ | 141.6 (107.1)^e^ | 118.3 (105.0) | 0.99 (0.989-0.995) | <.0001 | 0.99 (0.987-0.994) | <.0001 |

Data are n (%) or mean (standard deviation)

^§^ From the 337 patients, we have excluded all patients who were *lost to follow-up* (n = 14) and *not evaluated* (n = 18)*.*

^*^ SPP: smear positive pulmonary, SNP: smear negative pulmonary, EP: extra pulmonary.

TB: tuberculosis, SD: standard deviation.

^a^ 10 missing data, ^b^ 26 missing data, ^c^ 8 missing data, ^d^ 9 missing data, ^e^ 19 missing data. All missing data were imputed.
