# Supplementary material for: Factors Associated with Death during Tuberculosis Treatment of Patients Co-Infected with HIV at the Yaoundé Central Hospital, Cameroon: An 8-Year Hospital-Based Retrospective Cohort Study (2006–2013)
Source: PLoS One. 2014 Dec 15;9(12):e115211. doi: 10.1371/journal.pone.0115211 (PMC4266669; doi:10.1371/journal.pone.0115211)
Supplement: S2 Data — Data of the 337 patients included in the study. (PDF) [file pone.0115211.s002.pdf]

Data S2. Data of the 337 patients included in the study

N°	Year of TB diagnosis	Sex	Age (Years)	Last level education	Marital status	Area of habitation	Clinical presentation	Status of TB at diagnosis	Duration of known HIV infected (Years)	Body weight (Kgs)	Presence of another Comorbidity non-AIDS defining	Presence of AIDS defining disease (other than TB)	White Blood Cell Count (/mm3)	Hemoglobin level (g/dl)	Lymphocyte T4 count (/mm3)	Outcomes	Life duration during 6 months period follow-up (months)	Taking cotrimoxazole prophylactic therapy	Taking combined antiretroviral therapy
1	2006	Female	35	Secondary	Single	Urban	Extra Pulmonary TB only	New case	.0877	45	No	No	5400	7	12	Died	.03	Yes	Yes
2	2009	Female	30	Secondary	Single	Urban	Extra Pulmonary TB only	New case	.2521	52	No	Yes	23000	3	24	Died	.03	No	Yes
3	2009	Male	55	University	Married	Rural	Extra Pulmonary TB only	New case	.6356	53	No	No	6000	8	48	Died	.03	No	Yes
4	2013	Male	30	Primary	Single	Rural	Smear-negative Pulmonary TB only	New case	.0740	53	No	No	3900	9	125	Died	.03	No	Yes
5	2006	Female	37	University	Married	Urban	Smear-Positive Pulmonary TB only	New case	.0219	52	No	Yes	4200	4	2	Died	.03	Yes	Yes
6	2006	Female	50	Secondary	Widow	Urban	Extra Pulmonary TB only	New case	.0630	85	No	No	5800	9	12	Died	.07	Yes	Yes
7	2008	Female	48	Secondary	Single	Urban	Extra Pulmonary TB only	New case	.1644	47	No	No	14500	11	13	Died	.07	No	No
8	2009	Male	38	Secondary	Single	Urban	Extra Pulmonary TB only	New case	.0164	45	No	No	2300	8	42	Died	.07	Yes	Yes
9	2009	Female	37	Primary	Single	Urban	Extra Pulmonary TB only	New case	.6740	57	No	No	5500	12	120	Died	.07	Yes	Yes
10	2009	Female	31	University	Single	Urban	Extra Pulmonary TB only	New case	.0027	50	No	No	4100	8	172	Died	.07	Yes	No
11	2009	Male	50	Secondary	Married	Rural	Extra Pulmonary TB only	New case	1.7726	49	No	No	8700	8	500	Died	.07	No	No
12	2006	Female	34	Secondary	Single	Urban	Smear-negative Pulmonary TB only	New case	.0384	55	No	No	7360	6	146	Died	.07	Yes	Yes
13	2009	Female	30	Primary	Single	Rural	Smear-negative Pulmonary TB only	New case	.1589	40	No	Yes	2290	8	13	Died	.07	Yes	Yes
14	2007	Male	35	Primary	Single	Urban	Smear-Positive Pulmonary TB only	New case	.0329	39	No	No	3300	6	75	Died	.07	Yes	Yes
15	2010	Male	28	University	Single	Urban	Smear-Positive Pulmonary TB only	New case	.1205	60	No	No	3980	9	168	Died	.07	Yes	Yes
16	2012	Male	64	Secondary	Married	Urban	Smear-Positive Pulmonary TB only	New case	.0384	62	No	Yes	5150	11	251	Died	.07	No	No
17	2013	Female	34	Primary	Single	Urban	Smear-Positive Pulmonary TB only	New case	.0712	48	No	No	4860	8	156	Died	.07	Yes	Yes
18	2006	Female	26	No formal	Single	Urban	Extra Pulmonary TB only	New case	.0082	36	No	No	11000	7	14	Died	.10	No	No
19	2006	Female	25	Secondary	Single	Rural	Extra Pulmonary TB only	New case	.0438	44	Yes	No	9370	6	104	Died	.10	Yes	Yes
20	2011	Male	51	University	Single	Urban	Extra Pulmonary TB only	Retreatment case	1.5562	45	Yes	No	2300	7	12	Died	.10	Yes	Yes
21	2011	Male	58	University	Married	Urban	Extra Pulmonary TB only	New case	.0000	52	No	Yes	6000	8	41	Died	.10	Yes	No
22	2009	Male	42	Secondary	Single	Urban	Smear-negative Pulmonary TB only	New case	.1507	38	No	No	1600	6	4	Died	.10	Yes	Yes
23	2007	Female	33	University	Single	Rural	Smear-Positive Pulmonary TB only	New case	.0219	47	No	No	13900	2	59	Died	.10	Yes	No
24	2008	Female	35	Secondary	Single	Urban	Smear-Positive Pulmonary TB only	New case	.0219	42	No	No	11600	5	97	Died	.10	Yes	No
25	2011	Female	36	Secondary	Married	Rural	Smear-Positive Pulmonary TB only	New case	.3041	.	No	No	.	.	.	Died	.10	No	No
26	2007	Male	37	Primary	Married	Urban	Mixed Form	New case	.0548	62	No	Yes	10300	8	15	Died	.13	Yes	Yes
27	2006	Male	54	Secondary	Married	Urban	Extra Pulmonary TB only	Retreatment case	.0055	84	No	Yes	17000	8	4	Died	.13	No	Yes
28	2007	Female	27	No formal	Single	Urban	Extra Pulmonary TB only	New case	.2000	37	No	No	8300	2	34	Died	.13	Yes	Yes
29	2011	Female	28	University	Single	Urban	Extra Pulmonary TB only	New case	.0000	.	No	No	.	.	.	Died	.13	No	No
30	2010	Male	33	Primary	Married	Urban	Smear-Positive Pulmonary TB only	New case	.0055	48	No	No	31100	8	11	Died	.13	No	No
31	2013	Female	25	Primary	Single	Urban	Smear-Positive Pulmonary TB only	New case	.0301	72	No	No	4320	7	124	Died	.13	No	Yes
32	2008	Male	46	Secondary	Married	Urban	Mixed Form	Retreatment case	.0000	42	Yes	No	2300	5	42	Died	.17	No	No
33	2006	Male	33	Secondary	Single	Urban	Extra Pulmonary TB only	New case	.0000	49	No	Yes	3200	10	40	Died	.17	Yes	Yes
34	2006	Male	41	University	Married	Urban	Extra Pulmonary TB only	New case	.0192	63	No	No	3200	7	46	Died	.17	No	Yes
35	2007	Female	37	Secondary	Single	Urban	Extra Pulmonary TB only	New case	.1452	37	No	No	2700	9	25	Died	.17	Yes	No
36	2011	Female	42	No formal	Widow	Urban	Extra Pulmonary TB only	New case	.2137	50	No	No	1900	9	92	Died	.17	Yes	Yes
37	2006	Female	33	Secondary	Single	Urban	Smear-negative Pulmonary TB only	New case	.0301	41	Yes	No	8490	6	7	Died	.17	No	No
38	2007	Female	36	University	Single	Urban	Smear-Positive Pulmonary TB only	New case	.3781	64	No	No	6000	6	48	Died	.17	Yes	Yes
39	2009	Male	50	University	Married	Urban	Mixed Form	New case	.0000	78	Yes	Yes	2010	10	4	Died	.20	Yes	Yes
40	2007	Male	40	Secondary	Married	Urban	Extra Pulmonary TB only	New case	.0082	48	No	No	6600	4	181	Died	.20	Yes	Yes
41	2007	Male	34	No formal	Single	Urban	Extra Pulmonary TB only	New case	.0438	40	No	No	.	.	297	Died	.20	Yes	No
42	2009	Male	53	No formal	Single	Urban	Extra Pulmonary TB only	New case	.4329	48	No	No	2100	8	14	Died	.20	No	No
43	2012	Male	38	Primary	Married	Urban	Extra Pulmonary TB only	New case	.0055	52	Yes	No	9900	15	51	Died	.20	Yes	Yes
44	2008	Male	35	University	Single	Rural	Smear-negative Pulmonary TB only	New case	.0192	.	No	No	11100	11	45	Died	.20	Yes	No
45	2010	Female	78	Secondary	Widow	Urban	Smear-Positive Pulmonary TB only	New case	.0356	52	No	No	3260	10	49	Died	.20	No	No
46	2012	Male	48	Secondary	Married	Urban	Smear-Positive Pulmonary TB only	New case	.0000	30	Yes	No	3700	8	11	Died	.20	Yes	Yes
47	2009	Male	35	University	Single	Urban	Mixed Form	New case	.0904	.	No	No	4700	12	.	Died	.23	Yes	Yes
48	2013	Female	35	Secondary	Single	Urban	Extra Pulmonary TB only	New case	5.6603	38	No	No	29100	5	108	Died	.23	Yes	No
49	2006	Male	46	Secondary	Married	Urban	Extra Pulmonary TB only	New case	.1342	52	No	Yes	25000	9	275	Died	.27	No	Yes
50	2007	Male	29	No formal	Single	Rural	Extra Pulmonary TB only	New case	.1671	55	No	No	1000	5	37	Died	.27	Yes	Yes
51	2010	Male	45	University	Married	Urban	Smear-Positive Pulmonary TB only	Retreatment case	3.2767	63	No	No	3900	10	8	Died	.27	Yes	Yes
52	2009	Male	50	Secondary	Divorced	Urban	Smear-negative Pulmonary TB only	New case	.3096	50	No	No	900	9	174	Died	.30	Yes	Yes
53	2009	Female	65	Primary	Married	Urban	Extra Pulmonary TB only	New case	.0000	54	No	Yes	.	.	.	Died	.33	Yes	Yes
54	2008	Female	26	Secondary	Single	Urban	Smear-Positive Pulmonary TB only	New case	.3616	37	No	Yes	4000	6	6	Died	.33	Yes	Yes
55	2013	Male	53	Primary	Married	Urban	Smear-Positive Pulmonary TB only	New case	.8247	42	No	No	2430	6	27	Died	.33	Yes	Yes
56	2013	Female	51	Secondary	Widow	Urban	Smear-Positive Pulmonary TB only	Retreatment case	1.7315	43	Yes	No	1700	9	163	Died	.33	No	Yes
57	2007	Female	40	University	Married	Urban	Extra Pulmonary TB only	New case	.0247	55	No	Yes	8600	9	151	Died	.37	Yes	No
58	2006	Male	34	Secondary	Married	Urban	Extra Pulmonary TB only	New case	.0055	48	No	Yes	4910	10	9	Died	.40	Yes	No
59	2006	Male	50	University	Married	Urban	Smear-negative Pulmonary TB only	Retreatment case	1.6904	47	No	No	7550	8	48	Died	.40	Yes	Yes
60	2009	Male	43	Secondary	Married	Urban	Smear-negative Pulmonary TB only	New case	.0438	55	No	Yes	5610	12	138	Died	.40	Yes	No
61	2008	Male	44	Secondary	Married	Rural	Smear-negative Pulmonary TB only	Retreatment case	6.3315	48	No	No	8100	7	1	Died	.43	Yes	No
62	2008	Male	25	Secondary	Married	Rural	Smear-negative Pulmonary TB only	New case	.0301	56	No	No	3700	11	33	Died	.43	No	No
63	2006	Female	43	No formal	Widow	Urban	Smear-Positive Pulmonary TB only	New case	.0274	55	No	No	3000	5	15	Died	.43	Yes	Yes
64	2008	Male	39	Primary	Married	Urban	Extra Pulmonary TB only	New case	.0247	58	No	No	1500	6	56	Died	.47	Yes	Yes
65	2007	Male	43	Secondary	Widow	Urban	Extra Pulmonary TB only	New case	5.8110	38	No	No	1500	8	35	Died	.50	Yes	Yes
66	2007	Male	44	University	Married	Urban	Extra Pulmonary TB only	New case	.3288	45	No	No	2200	9	43	Died	.50	Yes	Yes
67	2013	Female	48	University	Married	Urban	Smear-negative Pulmonary TB only	Retreatment case	1.3836	56	Yes	No	3100	5	53	Died	.57	Yes	Yes
68	2006	Female	25	Secondary	Single	Rural	Extra Pulmonary TB only	New case	.0603	.	No	No	4200	7	187	Died	.60	No	No
69	2012	Male	52	University	Married	Urban	Extra Pulmonary TB only	New case	.1096	74	No	Yes	4080	8	25	Died	.60	No	Yes
70	2006	Female	35	Secondary	Single	Urban	Extra Pulmonary TB only	New case	.0000	48	No	No	3540	4	58	Died	.67	Yes	Yes
71	2010	Female	44	Primary	Married	Urban	Smear-negative Pulmonary TB only	New case	.0164	44	No	Yes	8000	3	50	Died	.70	No	No
72	2006	Male	52	University	Married	Urban	Extra Pulmonary TB only	New case	.0000	52	No	No	5400	6	281	Died	.77	No	No
73	2007	Female	46	No formal	Single	Urban	Smear-negative Pulmonary TB only	New case	.0000	39	Yes	No	4090	6	21	Died	.77	Yes	Yes
74	2011	Female	60	Primary	Married	Urban	Smear-negative Pulmonary TB only	Retreatment case	1.2356	54	Yes	Yes	54200	9	35	Died	.77	No	Yes
75	2006	Female	23	University	Single	Urban	Mixed Form	New case	.0658	42	No	Yes	630	5	22	Died	.83	Yes	No
76	2008	Female	45	University	Single	Urban	Smear-Positive Pulmonary TB only	New case	.0000	42	Yes	No	7800	8	42	Died	.90	No	No
77	2008	Male	24	Secondary	Single	Urban	Mixed Form	New case	.2959	.	No	No	.	.	.	Died	.97	No	No
78	2013	Female	28	Primary	Single	Urban	Smear-Positive Pulmonary TB only	New case	.0110	35	No	No	9100	8	69	Died	.97	No	Yes

79	2008	Male	41	Secondary	Married	Urban	Extra Pulmonary TB only	New case	.2658	60	No	No	800	7	96	Died	1.00	Yes	Yes
80	2007	Female	58	Secondary	Married	Urban	Smear-negative Pulmonary TB only	New case	1.0137	.	Yes	No	.	.	.	Died	1.10	Yes	No
81	2006	Female	32	No formal	Single	Urban	Smear-Positive Pulmonary TB only	New case	.0000	41	Yes	No	4900	12	44	Died	1.10	Yes	Yes
82	2008	Female	27	Secondary	Married	Urban	Smear-Positive Pulmonary TB only	Retreatment case	.0110	28	No	Yes	6100	7	17	Died	1.10	No	Yes
83	2007	Male	51	Primary	Single	Urban	Smear-Positive Pulmonary TB only	New case	.0082	.	No	Yes	4700	13	14	Died	1.37	Yes	Yes
84	2008	Male	32	No formal	Single	Urban	Smear-Positive Pulmonary TB only	New case	.0137	45	No	No	6600	7	23	Died	1.40	Yes	Yes
85	2013	Male	40	Primary	Single	Urban	Extra Pulmonary TB only	New case	.0000	73	No	No	3250	12	23	Died	1.50	No	No
86	2009	Male	28	Primary	Married	Urban	Smear-Positive Pulmonary TB only	New case	.3890	.	No	No	.	.	.	Died	1.63	Yes	Yes
87	2007	Male	33	Secondary	Single	Urban	Smear-Positive Pulmonary TB only	New case	.0027	61	No	Yes	1700	11	20	Died	1.90	Yes	No
88	2008	Male	33	University	Single	Urban	Smear-negative Pulmonary TB only	New case	.0110	42	Yes	Yes	3800	10	31	Died	2.00	Yes	Yes
89	2008	Female	26	Secondary	Single	Rural	Smear-Positive Pulmonary TB only	New case	.0329	52	No	No	12900	12	22	Died	2.03	Yes	No
90	2009	Male	22	Secondary	Single	Urban	Smear-Positive Pulmonary TB only	New case	1.9616	59	Yes	No	3500	8	14	Died	2.10	Yes	Yes
91	2008	Male	35	Secondary	Single	Urban	Mixed Form	New case	.1123	65	No	Yes	6000	9	193	Died	2.13	Yes	No
92	2011	Female	50	University	Widow	Urban	Smear-Positive Pulmonary TB only	New case	.0384	32	Yes	No	3300	6	25	Died	2.20	No	Yes
93	2009	Female	20	Primary	Single	Urban	Smear-Positive Pulmonary TB only	New case	.0110	43	Yes	Yes	10000	7	9	Died	2.43	Yes	Yes
94	2013	Female	48	No formal	Single	Urban	Extra Pulmonary TB only	Retreatment case	.0000	35	Yes	No	6400	8	75	Died	2.50	Yes	No
95	2008	Female	26	Secondary	Single	Urban	Smear-Positive Pulmonary TB only	New case	.0082	58	No	No	15000	5	32	Died	2.97	Yes	Yes
96	2013	Male	39	Secondary	Single	Urban	Mixed Form	New case	.9863	39	No	No	2180	9	6	Died	3.20	Yes	Yes
97	2008	Female	36	University	Married	Urban	Smear-negative Pulmonary TB only	New case	.0082	60	Yes	Yes	900	7	162	Died	3.30	Yes	Yes
98	2013	Male	52	University	Married	Urban	Extra Pulmonary TB only	New case	7.1260	45	No	No	5400	10	54	Died	3.67	No	Yes
99	2010	Male	37	University	Married	Urban	Smear-negative Pulmonary TB only	New case	.0466	44	No	Yes	2500	8	143	Died	5.07	Yes	No
100	2007	Female	24	Secondary	Single	Urban	Mixed Form	New case	.0000	39	No	No	1990	6	49	Not evaluated	6.00	Yes	Yes
101	2007	Female	24	Secondary	Single	Urban	Extra Pulmonary TB only	New case	.1288	42	No	No	7000	8	140	Not evaluated	6.00	Yes	Yes
102	2008	Male	33	Secondary	Single	Urban	Extra Pulmonary TB only	New case	.0082	70	No	No	4700	6	350	Not evaluated	6.00	No	No
103	2009	Female	45	Secondary	Single	Rural	Extra Pulmonary TB only	New case	.1342	47	No	No	7200	4	145	Not evaluated	6.00	Yes	Yes
104	2009	Male	31	No formal	Married	Urban	Extra Pulmonary TB only	New case	.0301	63	No	No	6900	11	294	Not evaluated	6.00	Yes	Yes
105	2010	Male	42	No formal	Married	Urban	Extra Pulmonary TB only	New case	.0959	60	No	No	5100	10	64	Not evaluated	6.00	Yes	No
106	2010	Female	38	Secondary	Married	Urban	Extra Pulmonary TB only	New case	4.3288	67	No	No	11100	10	168	Not evaluated	6.00	Yes	Yes
107	2011	Male	45	Secondary	Married	Urban	Extra Pulmonary TB only	New case	.0000	58	No	No	4400	7	28	Not evaluated	6.00	Yes	Yes
108	2006	Male	54	University	Married	Urban	Smear-negative Pulmonary TB only	New case	.0219	63	Yes	No	11400	8	3	Not evaluated	6.00	Yes	Yes
109	2006	Female	52	Secondary	Widow	Urban	Smear-negative Pulmonary TB only	Retreatment case	1.3973	60	No	No	8200	7	170	Not evaluated	6.00	Yes	Yes
110	2006	Male	39	Primary	Married	Rural	Smear-negative Pulmonary TB only	New case	.0384	63	No	No	8800	14	304	Not evaluated	6.00	Yes	No
111	2006	Female	55	No formal	Widow	Urban	Smear-Positive Pulmonary TB only	New case	.0740	55	No	No	7760	11	128	Not evaluated	6.00	Yes	Yes
112	2006	Female	45	University	Married	Urban	Smear-Positive Pulmonary TB only	New case	.0000	56	No	No	7050	7	174	Not evaluated	6.00	Yes	No
113	2007	Male	25	University	Single	Urban	Smear-Positive Pulmonary TB only	New case	2.1808	60	Yes	Yes	20000	9	62	Not evaluated	6.00	Yes	Yes
114	2008	Female	35	University	Single	Urban	Smear-Positive Pulmonary TB only	New case	.0932	58	No	No	.	.	.	Not evaluated	6.00	Yes	No
115	2008	Female	33	Secondary	Married	Urban	Smear-Positive Pulmonary TB only	New case	.0000	65	No	No	2800	10	237	Not evaluated	6.00	Yes	Yes
116	2009	Female	38	Secondary	Single	Urban	Smear-Positive Pulmonary TB only	New case	.0000	56	No	No	400	3	124	Not evaluated	6.00	Yes	No
117	2012	Female	63	No formal	Married	Urban	Smear-Positive Pulmonary TB only	New case	.0027	56	No	No	5000	12	56	Not evaluated	6.00	No	No
118	2007	Female	41	No formal	Single	Urban	Mixed Form	New case	.7397	40	No	Yes	2500	12	100	Lost to follow-up	6.00	Yes	Yes
119	2013	Female	28	No formal	Single	Urban	Mixed Form	New case	.6849	60	No	No	8300	9	175	Lost to follow-up	6.00	No	No
120	2006	Female	26	Secondary	Single	Urban	Extra Pulmonary TB only	New case	.0082	60	No	No	12900	8	166	Lost to follow-up	6.00	Yes	Yes
121	2006	Female	44	Secondary	Married	Urban	Extra Pulmonary TB only	New case	.4247	50	No	No	7000	9	617	Lost to follow-up	6.00	No	Yes
122	2006	Female	35	Secondary	Single	Urban	Smear-negative Pulmonary TB only	New case	.0274	52	No	No	12800	7	193	Lost to follow-up	6.00	No	No
123	2007	Female	39	No formal	Married	Urban	Smear-negative Pulmonary TB only	New case	.0192	52	No	Yes	5700	16	6	Lost to follow-up	6.00	Yes	Yes
124	2007	Male	35	Primary	Married	Urban	Smear-Positive Pulmonary TB only	New case	.0055	51	No	No	6200	9	2	Lost to follow-up	6.00	Yes	Yes
125	2007	Female	37	No formal	Single	Urban	Smear-Positive Pulmonary TB only	Retreatment case	.0000	.	No	No	3940	9	29	Lost to follow-up	6.00	Yes	Yes
126	2008	Female	20	Secondary	Married	Urban	Smear-Positive Pulmonary TB only	New case	.0548	58	No	No	.	.	.	Lost to follow-up	6.00	Yes	Yes
127	2008	Female	34	Secondary	Single	Urban	Smear-Positive Pulmonary TB only	New case	.0055	62	No	No	10700	8	13	Lost to follow-up	6.00	Yes	No
128	2008	Female	29	University	Single	Urban	Smear-Positive Pulmonary TB only	New case	.0110	47	No	No	6300	5	48	Lost to follow-up	6.00	Yes	No
129	2009	Female	46	Secondary	Single	Urban	Smear-Positive Pulmonary TB only	New case	3.9068	40	No	No	8400	8	178	Lost to follow-up	6.00	Yes	Yes
130	2009	Male	42	Secondary	Single	Urban	Smear-Positive Pulmonary TB only	New case	.1726	52	Yes	No	6200	9	375	Lost to follow-up	6.00	No	No
131	2010	Female	38	Secondary	Single	Urban	Smear-Positive Pulmonary TB only	New case	.0493	35	No	No	1200	5	5	Lost to follow-up	6.00	Yes	No
132	2006	Male	36	Secondary	Single	Urban	Smear-Positive Pulmonary TB only	Retreatment case	.8027	.	No	No	3400	9	154	Failed	6.00	Yes	Yes
133	2007	Male	37	Secondary	Single	Urban	Mixed Form	New case	1.0219	58	No	Yes	5700	9	417	Cured	6.00	Yes	Yes
134	2010	Male	35	Secondary	Single	Urban	Mixed Form	New case	.0219	50	No	No	9500	8	24	Cured	6.00	Yes	Yes
135	2006	Female	42	Secondary	Married	Urban	Extra Pulmonary TB only	New case	.1808	70	No	No	8000	7	116	Cured	6.00	No	Yes
136	2006	Female	38	Secondary	Married	Urban	Extra Pulmonary TB only	New case	1.2137	47	No	No	3400	8	215	Cured	6.00	Yes	No
137	2006	Male	46	Secondary	Married	Urban	Extra Pulmonary TB only	New case	.1945	52	Yes	Yes	25000	9	275	Cured	6.00	No	Yes
138	2007	Male	30	Primary	Married	Urban	Extra Pulmonary TB only	New case	.0000	46	No	No	.	.	12	Cured	6.00	Yes	Yes
139	2007	Male	45	No formal	Single	Rural	Extra Pulmonary TB only	New case	5.0575	68	No	No	3700	8	267	Cured	6.00	Yes	No
140	2008	Female	35	University	Single	Urban	Extra Pulmonary TB only	Retreatment case	8.5205	49	No	No	3750	9	74	Cured	6.00	Yes	Yes
141	2008	Male	48	University	Married	Urban	Extra Pulmonary TB only	New case	.2521	58	Yes	No	11700	7	79	Cured	6.00	Yes	Yes
142	2008	Female	26	Secondary	Single	Urban	Extra Pulmonary TB only	New case	.2630	44	No	No	4360	8	201	Cured	6.00	Yes	Yes
143	2009	Male	49	Secondary	Married	Rural	Extra Pulmonary TB only	New case	.0164	50	No	No	6400	11	146	Cured	6.00	Yes	Yes
144	2010	Female	30	No formal	Single	Urban	Extra Pulmonary TB only	New case	.0219	39	No	No	6100	5	128	Cured	6.00	No	Yes
145	2011	Female	32	Secondary	Married	Urban	Extra Pulmonary TB only	New case	.3370	64	No	No	2000	3	.	Cured	6.00	No	Yes
146	2011	Male	33	University	Single	Rural	Extra Pulmonary TB only	New case	5.1479	46	No	No	4250	8	306	Cured	6.00	Yes	Yes
147	2013	Male	57	Primary	Married	Urban	Extra Pulmonary TB only	Retreatment case	4.5726	51	No	No	6100	7	13	Cured	6.00	No	Yes
148	2013	Female	27	University	Single	Urban	Extra Pulmonary TB only	New case	.2548	65	No	No	5300	7	187	Cured	6.00	Yes	No
149	2006	Female	43	No formal	Married	Urban	Smear-negative Pulmonary TB only	New case	.0000	45	No	No	3290	8	14	Cured	6.00	Yes	Yes
150	2006	Male	26	Primary	Single	Urban	Smear-negative Pulmonary TB only	New case	.7863	60	No	No	3000	8	31	Cured	6.00	Yes	Yes
151	2006	Female	34	University	Single	Urban	Smear-negative Pulmonary TB only	New case	.0110	45	No	No	9320	8	132	Cured	6.00	Yes	Yes
152	2006	Male	35	Secondary	Married	Urban	Smear-negative Pulmonary TB only	New case	.0164	.	No	No	4660	8	415	Cured	6.00	Yes	Yes
153	2008	Female	27	Secondary	Single	Urban	Smear-negative Pulmonary TB only	New case	.0110	.	Yes	No	5000	6	9	Cured	6.00	Yes	Yes
154	2009	Female	30	Secondary	Single	Urban	Smear-negative Pulmonary TB only	New case	.0000	65	No	No	6600	5	238	Cured	6.00	Yes	No
155	2010	Female	57	Primary	Married	Urban	Smear-negative Pulmonary TB only	New case	.0082	58	No	No	3000	8	195	Cured	6.00	Yes	No
156	2011	Male	49	University	Married	Urban	Smear-negative Pulmonary TB only	New case	2.5041	67	No	Yes	3170	6	42	Cured	6.00	No	Yes
157	2006	Male	47	No formal	Married	Urban	Smear-Positive Pulmonary TB only	New case	2.2466	58	Yes	No	5000	11	8	Cured	6.00	Yes	Yes
158	2006	Male	40	Secondary	Married	Urban	Smear-Positive Pulmonary TB only	New case	.0164	50	No	No	2130	9	33	Cured	6.00	Yes	Yes
159	2006	Female	44	Secondary	Widow	Urban	Smear-Positive Pulmonary TB only	New case	.1041	47	No	No	2700	9	35	Cured	6.00	Yes	Yes
160	2006	Female	27	Secondary	Married	Urban	Smear-Positive Pulmonary TB only	New case	.0000	38	No	No	3000	10	137	Cured	6.00	Yes	No
161	2006	Male	40	Secondary	Married	Urban	Smear-Positive Pulmonary TB only	New case	.9534	52	Yes	No	4300	8	155	Cured	6.00	Yes	Yes
162	2006	Male	32	Primary	Married	Urban	Smear-Positive Pulmonary TB only	New case	.0027	56	No	No	9500	9	261	Cured	6.00	No	No
163	2007	Male	39	Secondary	Single	Urban	Smear-Positive Pulmonary TB only	New case	.0137	.	No	Yes	18500	7	.	Cured	6.00	Yes	Yes

164	2007	Male	46	Secondary	Married	Urban	Smear-Positive Pulmonary TB only	New case	1.2137	57	No	No	2900	10	60	Cured	6.00	Yes	Yes
165	2007	Male	47	Secondary	Married	Urban	Smear-Positive Pulmonary TB only	Retreatment case	.5041	65	No	No	4800	10	125	Cured	6.00	Yes	Yes
166	2007	Female	37	No formal	Single	Urban	Smear-Positive Pulmonary TB only	Retreatment case	1.9726	60	No	No	7990	11	189	Cured	6.00	Yes	Yes
167	2007	Male	36	Secondary	Single	Urban	Smear-Positive Pulmonary TB only	New case	.3973	45	No	No	5000	7	193	Cured	6.00	Yes	Yes
168	2007	Female	35	Primary	Married	Rural	Smear-Positive Pulmonary TB only	Retreatment case	.0247	60	No	No	4700	10	383	Cured	6.00	Yes	No
169	2008	Male	36	Secondary	Single	Urban	Smear-Positive Pulmonary TB only	New case	.0000	45	No	No	4600	11	132	Cured	6.00	Yes	No
170	2008	Female	26	Secondary	Single	Urban	Smear-Positive Pulmonary TB only	New case	.0795	.	No	No	.	.	154	Cured	6.00	Yes	Yes
171	2008	Male	33	Secondary	Single	Urban	Smear-Positive Pulmonary TB only	New case	-.0027	65	No	No	6600	12	309	Cured	6.00	Yes	No
172	2009	Female	59	No formal	Married	Urban	Smear-Positive Pulmonary TB only	New case	.0658	53	No	No	4100	5	109	Cured	6.00	Yes	Yes
173	2009	Female	28	Primary	Single	Urban	Smear-Positive Pulmonary TB only	New case	.1534	54	No	No	4500	9	214	Cured	6.00	Yes	Yes
174	2009	Female	62	Secondary	Married	Urban	Smear-Positive Pulmonary TB only	New case	.0219	58	No	No	3000	10	229	Cured	6.00	Yes	No
175	2009	Female	29	Primary	Single	Rural	Smear-Positive Pulmonary TB only	New case	.1397	50	No	No	5800	6	263	Cured	6.00	Yes	Yes
176	2009	Female	18	Secondary	Single	Urban	Smear-Positive Pulmonary TB only	New case	.0110	48	No	No	12900	8	302	Cured	6.00	No	No
177	2009	Male	45	Secondary	Married	Urban	Smear-Positive Pulmonary TB only	New case	2.0329	54	No	No	2900	10	408	Cured	6.00	Yes	Yes
178	2010	Male	52	University	Married	Urban	Smear-Positive Pulmonary TB only	New case	.0055	68	No	No	8000	12	4	Cured	6.00	Yes	Yes
179	2010	Male	40	No formal	Married	Urban	Smear-Positive Pulmonary TB only	New case	.0219	60	No	No	3400	7	102	Cured	6.00	Yes	Yes
180	2010	Female	46	University	Married	Urban	Smear-Positive Pulmonary TB only	New case	.0356	72	No	No	5600	12	102	Cured	6.00	Yes	Yes
181	2010	Female	47	No formal	Widow	Rural	Smear-Positive Pulmonary TB only	New case	2.6959	42	No	No	4400	8	129	Cured	6.00	Yes	Yes
182	2010	Female	20	Secondary	Single	Urban	Smear-Positive Pulmonary TB only	Retreatment case	.0027	59	No	No	5920	10	165	Cured	6.00	Yes	No
183	2010	Male	36	Secondary	Single	Urban	Smear-Positive Pulmonary TB only	Retreatment case	7.7233	54	No	No	8300	4	183	Cured	6.00	Yes	Yes
184	2010	Female	33	University	Single	Urban	Smear-Positive Pulmonary TB only	New case	3.4192	52	No	No	2440	7	216	Cured	6.00	Yes	No
185	2010	Male	22	Primary	Single	Rural	Smear-Positive Pulmonary TB only	New case	.0082	54	No	No	9030	8	343	Cured	6.00	Yes	Yes
186	2011	Male	43	Primary	Married	Urban	Smear-Positive Pulmonary TB only	New case	.0192	.	No	No	.	.	.	Cured	6.00	Yes	No
187	2011	Female	24	Secondary	Single	Urban	Smear-Positive Pulmonary TB only	New case	.4192	.	No	No	4500	8	.	Cured	6.00	Yes	Yes
188	2011	Male	40	University	Single	Urban	Smear-Positive Pulmonary TB only	Retreatment case	1.1671	53	No	Yes	6410	11	16	Cured	6.00	Yes	Yes
189	2011	Female	40	Secondary	Married	Urban	Smear-Positive Pulmonary TB only	New case	.5041	43	No	No	1900	7	24	Cured	6.00	Yes	Yes
190	2011	Female	37	No formal	Single	Urban	Smear-Positive Pulmonary TB only	New case	.1644	.	No	No	3440	12	37	Cured	6.00	Yes	No
191	2012	Female	32	Secondary	Married	Urban	Smear-Positive Pulmonary TB only	New case	.0822	72	No	No	.	.	.	Cured	6.00	Yes	Yes
192	2012	Male	47	University	Married	Rural	Smear-Positive Pulmonary TB only	Retreatment case	.1068	55	No	No	1400	6	8	Cured	6.00	Yes	Yes
193	2012	Female	42	University	Married	Urban	Smear-Positive Pulmonary TB only	Retreatment case	.1260	48	No	No	5500	9	41	Cured	6.00	Yes	Yes
194	2012	Male	68	Secondary	Married	Urban	Smear-Positive Pulmonary TB only	New case	.0274	53	No	No	22800	7	86	Cured	6.00	Yes	Yes
195	2012	Male	35	University	Married	Urban	Smear-Positive Pulmonary TB only	Retreatment case	.0959	59	Yes	No	8500	13	159	Cured	6.00	Yes	Yes
196	2012	Female	52	Secondary	Married	Urban	Smear-Positive Pulmonary TB only	New case	.9973	52	Yes	No	10200	11	183	Cured	6.00	Yes	Yes
197	2012	Female	25	Secondary	Single	Urban	Smear-Positive Pulmonary TB only	New case	.2082	58	No	No	5300	7	187	Cured	6.00	Yes	Yes
198	2012	Female	39	University	Married	Urban	Smear-Positive Pulmonary TB only	New case	.0658	39	No	No	3700	7	192	Cured	6.00	Yes	No
199	2013	Female	47	Primary	Widow	Urban	Smear-Positive Pulmonary TB only	New case	9.6027	58	No	No	5800	13	97	Cured	6.00	Yes	Yes
200	2013	Male	60	University	Married	Urban	Smear-Positive Pulmonary TB only	New case	.4849	53	No	No	6000	9	114	Cured	6.00	Yes	Yes
201	2013	Female	19	Primary	Single	Urban	Smear-Positive Pulmonary TB only	New case	.0000	52	No	Yes	14000	12	196	Cured	6.00	No	No
202	2013	Male	28	Secondary	Married	Rural	Smear-Positive Pulmonary TB only	New case	-.0027	65	No	No	4350	13	294	Cured	6.00	Yes	Yes
203	2013	Female	54	Secondary	Married	Urban	Smear-Positive Pulmonary TB only	New case	.0247	54	No	No	4090	13	302	Cured	6.00	Yes	No
204	2007	Female	44	Primary	Single	Urban	Mixed Form	New case	.0027	61	No	Yes	10400	6	79	Completed treatment	6.00	Yes	Yes
205	2007	Male	52	University	Single	Urban	Mixed Form	New case	.1753	58	Yes	No	2170	10	132	Completed treatment	6.00	Yes	Yes
206	2009	Female	36	Secondary	Single	Urban	Mixed Form	New case	.0767	48	No	Yes	12900	6	50	Completed treatment	6.00	No	Yes
207	2009	Male	52	University	Married	Urban	Mixed Form	New case	.0521	74	No	No	3900	7	222	Completed treatment	6.00	Yes	Yes
208	2013	Male	42	Secondary	Married	Rural	Mixed Form	Retreatment case	8.6247	63	No	No	4200	8	46	Completed treatment	6.00	Yes	Yes
209	2006	Male	40	Primary	Married	Urban	Extra Pulmonary TB only	New case	.0767	60	No	No	5700	9	9	Completed treatment	6.00	Yes	Yes
210	2006	Male	42	Secondary	Married	Urban	Extra Pulmonary TB only	New case	.5151	47	No	No	3200	11	32	Completed treatment	6.00	Yes	Yes
211	2006	Male	28	Secondary	Married	Rural	Extra Pulmonary TB only	New case	3.2082	.	No	No	7800	9	36	Completed treatment	6.00	Yes	Yes
212	2006	Female	49	University	Divorced	Urban	Extra Pulmonary TB only	New case	.4822	68	No	No	10200	12	105	Completed treatment	6.00	Yes	Yes
213	2006	Male	40	University	Married	Urban	Extra Pulmonary TB only	New case	1.9178	.	No	No	2000	10	164	Completed treatment	6.00	Yes	Yes
214	2006	Female	38	No formal	Widow	Urban	Extra Pulmonary TB only	New case	.1233	58	No	Yes	2960	6	187	Completed treatment	6.00	Yes	Yes
215	2007	Male	45	Secondary	Married	Urban	Extra Pulmonary TB only	New case	1.0027	.	No	Yes	5700	10	.	Completed treatment	6.00	Yes	Yes
216	2007	Female	42	Secondary	Divorced	Urban	Extra Pulmonary TB only	New case	.2795	45	No	Yes	13100	6	.	Completed treatment	6.00	Yes	Yes
217	2007	Male	39	Secondary	Married	Urban	Extra Pulmonary TB only	New case	.0247	39	No	No	13900	6	39	Completed treatment	6.00	Yes	Yes
218	2007	Male	45	Secondary	Married	Urban	Extra Pulmonary TB only	New case	.0110	.	No	Yes	9200	7	178	Completed treatment	6.00	Yes	Yes
219	2007	Male	42	University	Married	Urban	Extra Pulmonary TB only	Retreatment case	1.1342	60	No	No	2880	8	198	Completed treatment	6.00	Yes	Yes
220	2008	Female	33	No formal	Single	Rural	Extra Pulmonary TB only	New case	.1123	42	No	No	7600	8	98	Completed treatment	6.00	Yes	Yes
221	2008	Male	40	Secondary	Married	Urban	Extra Pulmonary TB only	New case	.0521	54	No	No	7400	9	102	Completed treatment	6.00	Yes	Yes
222	2008	Female	36	Secondary	Single	Rural	Extra Pulmonary TB only	New case	.0000	44	No	No	3600	8	108	Completed treatment	6.00	Yes	Yes
223	2008	Female	37	Secondary	Married	Urban	Extra Pulmonary TB only	Retreatment case	.1808	48	Yes	No	4300	9	138	Completed treatment	6.00	Yes	Yes
224	2008	Male	45	Primary	Married	Urban	Extra Pulmonary TB only	Retreatment case	.2712	42	No	No	5800	11	177	Completed treatment	6.00	No	No
225	2008	Male	40	No formal	Single	Urban	Extra Pulmonary TB only	New case	.1479	49	No	No	3900	8	361	Completed treatment	6.00	Yes	No
226	2009	Female	28	Secondary	Single	Urban	Extra Pulmonary TB only	New case	.0274	32	No	No	4800	8	73	Completed treatment	6.00	Yes	No
227	2009	Male	40	University	Married	Urban	Extra Pulmonary TB only	New case	.0712	42	No	No	4400	10	108	Completed treatment	6.00	Yes	Yes
228	2009	Male	58	Secondary	Married	Urban	Extra Pulmonary TB only	New case	.0219	57	No	No	5000	10	197	Completed treatment	6.00	Yes	Yes
229	2009	Male	50	Secondary	Married	Urban	Extra Pulmonary TB only	New case	.0795	60	No	No	9400	9	209	Completed treatment	6.00	Yes	Yes
230	2009	Male	33	Secondary	Single	Urban	Extra Pulmonary TB only	New case	.8411	70	No	No	5000	14	250	Completed treatment	6.00	Yes	No
231	2009	Male	35	University	Married	Urban	Extra Pulmonary TB only	New case	.0712	68	No	Yes	5000	4	415	Completed treatment	6.00	No	No
232	2009	Male	51	University	Married	Urban	Extra Pulmonary TB only	New case	.2575	60	No	Yes	7900	11	424	Completed treatment	6.00	Yes	No
233	2010	Female	33	Secondary	Single	Urban	Extra Pulmonary TB only	New case	.0849	68	No	No	2700	8	15	Completed treatment	6.00	Yes	Yes
234	2010	Male	39	University	Married	Urban	Extra Pulmonary TB only	Retreatment case	1.1014	50	No	No	7400	4	49	Completed treatment	6.00	Yes	Yes
235	2010	Female	42	University	Married	Urban	Extra Pulmonary TB only	New case	-.0027	65	No	No	5600	11	56	Completed treatment	6.00	Yes	Yes
236	2010	Male	51	Secondary	Married	Urban	Extra Pulmonary TB only	New case	.1534	64	No	No	2400	11	64	Completed treatment	6.00	Yes	Yes
237	2010	Female	32	No formal	Widow	Urban	Extra Pulmonary TB only	New case	.0055	53	No	No	3600	10	69	Completed treatment	6.00	Yes	Yes
238	2010	Male	40	University	Divorced	Urban	Extra Pulmonary TB only	New case	-.0027	55	No	No	7400	7	95	Completed treatment	6.00	Yes	Yes
239	2010	Female	31	University	Single	Urban	Extra Pulmonary TB only	New case	.0548	38	No	No	1400	8	109	Completed treatment	6.00	Yes	Yes
240	2010	Male	39	University	Single	Rural	Extra Pulmonary TB only	New case	.0795	71	No	No	5800	10	132	Completed treatment	6.00	Yes	Yes
241	2010	Male	47	Primary	Married	Rural	Extra Pulmonary TB only	New case	.0356	47	Yes	No	7000	10	144	Completed treatment	6.00	Yes	Yes
242	2010	Female	31	Secondary	Single	Urban	Extra Pulmonary TB only	Retreatment case	2.3726	48	No	No	11100	6	167	Completed treatment	6.00	Yes	Yes
243	2010	Male	40	Primary	Married	Urban	Extra Pulmonary TB only	New case	.8959	55	No	No	4800	9	194	Completed treatment	6.00	Yes	Yes
244	2010	Male	40	Secondary	Married	Urban	Extra Pulmonary TB only	Retreatment case	1.1973	45	No	No	4500	3	231	Completed treatment	6.00	Yes	No
245	2010	Male	55	No formal	Widow	Urban	Extra Pulmonary TB only	Retreatment case	.0055	58	No	No	8900	10	240	Completed treatment	6.00	No	No
246	2010	Male	52	University	Married	Rural	Extra Pulmonary TB only	New case	.0795	47	No	No	9400	10	244	Completed treatment	6.00	Yes	No
247	2011	Female	31	University	Single	Urban	Extra Pulmonary TB only	New case	.2110	48	No	No	5270	7	18	Completed treatment	6.00	Yes	Yes
248	2011	Female	32	University	Married	Urban	Extra Pulmonary TB only	New case	.0000	67	No	No	4560	9	185	Completed treatment	6.00	Yes	No

249	2011	Male	38	No formal	Married	Urban	Extra Pulmonary TB only	New case	.0384	70	No	No	5600	7	208	Completed treatment	6.00	Yes	Yes
250	2012	Female	38	Secondary	Married	Urban	Extra Pulmonary TB only	New case	.0000	54	No	No	.	.	.	Completed treatment	6.00	Yes	No
251	2012	Male	31	No formal	Single	Urban	Extra Pulmonary TB only	New case	.0082	54	No	No	9500	11	12	Completed treatment	6.00	Yes	Yes
252	2012	Male	31	No formal	Single	Urban	Extra Pulmonary TB only	New case	.0137	54	No	No	9500	11	12	Completed treatment	6.00	Yes	Yes
253	2012	Female	44	University	Single	Urban	Extra Pulmonary TB only	New case	.0137	60	No	No	2120	10	93	Completed treatment	6.00	Yes	No
254	2012	Female	36	No formal	Single	Urban	Extra Pulmonary TB only	New case	3.3918	54	No	No	11400	5	123	Completed treatment	6.00	No	Yes
255	2012	Female	43	University	Married	Urban	Extra Pulmonary TB only	New case	.0356	40	No	No	3100	7	142	Completed treatment	6.00	No	No
256	2012	Female	30	Primary	Divorced	Urban	Extra Pulmonary TB only	New case	.0411	65	No	No	3400	9	142	Completed treatment	6.00	Yes	Yes
257	2012	Male	38	Secondary	Married	Urban	Extra Pulmonary TB only	New case	5.0795	51	No	No	4950	6	153	Completed treatment	6.00	Yes	No
258	2012	Female	37	Secondary	Married	Urban	Extra Pulmonary TB only	New case	.2658	44	No	No	14500	8	156	Completed treatment	6.00	Yes	No
259	2012	Female	37	Primary	Married	Urban	Extra Pulmonary TB only	New case	.1233	55	No	No	19500	9	164	Completed treatment	6.00	No	Yes
260	2012	Female	42	Secondary	Single	Urban	Extra Pulmonary TB only	Retreatment case	.0082	54	No	No	4250	9	213	Completed treatment	6.00	Yes	Yes
261	2012	Male	53	Secondary	Married	Urban	Extra Pulmonary TB only	New case	2.8110	48	No	No	2100	8	341	Completed treatment	6.00	Yes	No
262	2013	Female	42	University	Married	Urban	Extra Pulmonary TB only	New case	.0082	46	No	No	3200	8	114	Completed treatment	6.00	Yes	Yes
263	2013	Female	31	Secondary	Single	Urban	Extra Pulmonary TB only	Retreatment case	.0000	57	No	No	5700	11	125	Completed treatment	6.00	Yes	Yes
264	2006	Female	48	No formal	Widow	Urban	Smear-negative Pulmonary TB only	New case	.0000	65	No	No	6200	10	19	Completed treatment	6.00	Yes	Yes
265	2006	Male	47	Primary	Single	Urban	Smear-negative Pulmonary TB only	New case	.1014	38	No	Yes	6440	6	40	Completed treatment	6.00	Yes	Yes
266	2006	Female	28	Secondary	Married	Rural	Smear-negative Pulmonary TB only	New case	.9890	40	No	Yes	12100	4	78	Completed treatment	6.00	No	No
267	2006	Male	46	University	Married	Urban	Smear-negative Pulmonary TB only	New case	.1151	72	No	No	7780	9	100	Completed treatment	6.00	Yes	Yes
268	2006	Male	44	University	Married	Rural	Smear-negative Pulmonary TB only	New case	.8493	.	No	No	13000	9	180	Completed treatment	6.00	Yes	Yes
269	2006	Female	43	Secondary	Married	Urban	Smear-negative Pulmonary TB only	New case	.0192	55	No	No	6800	11	255	Completed treatment	6.00	Yes	No
270	2006	Female	43	Secondary	Married	Urban	Smear-negative Pulmonary TB only	New case	.4904	52	No	Yes	2270	9	273	Completed treatment	6.00	Yes	Yes
271	2006	Female	52	Secondary	Divorced	Urban	Smear-negative Pulmonary TB only	New case	.0082	68	No	No	7900	11	500	Completed treatment	6.00	Yes	No
272	2007	Female	33	University	Single	Urban	Smear-negative Pulmonary TB only	New case	.0110	65	No	Yes	8000	6	.	Completed treatment	6.00	Yes	Yes
273	2007	Female	44	No formal	Married	Urban	Smear-negative Pulmonary TB only	New case	.0932	42	No	Yes	3900	8	89	Completed treatment	6.00	Yes	Yes
274	2007	Female	42	University	Married	Urban	Smear-negative Pulmonary TB only	New case	.1205	62	No	No	4200	9	177	Completed treatment	6.00	Yes	Yes
275	2007	Female	35	Secondary	Single	Urban	Smear-negative Pulmonary TB only	New case	.0027	65	No	No	8400	7	389	Completed treatment	6.00	Yes	No
276	2008	Female	45	Primary	Married	Urban	Smear-negative Pulmonary TB only	New case	.5973	.	No	No	.	.	.	Completed treatment	6.00	Yes	No
277	2008	Female	48	No formal	Widow	Rural	Smear-negative Pulmonary TB only	New case	.0877	.	No	No	1300	5	.	Completed treatment	6.00	Yes	Yes
278	2008	Male	43	Secondary	Married	Urban	Smear-negative Pulmonary TB only	Retreatment case	.8055	61	No	No	6100	10	21	Completed treatment	6.00	Yes	Yes
279	2008	Male	30	University	Single	Urban	Smear-negative Pulmonary TB only	New case	.4192	.	No	No	1800	6	77	Completed treatment	6.00	Yes	No
280	2008	Male	50	Secondary	Single	Urban	Smear-negative Pulmonary TB only	New case	.4192	64	No	No	5200	10	197	Completed treatment	6.00	No	Yes
281	2008	Female	31	University	Married	Urban	Smear-negative Pulmonary TB only	New case	3.6603	39	Yes	No	5600	7	200	Completed treatment	6.00	Yes	Yes
282	2008	Female	60	Secondary	Married	Urban	Smear-negative Pulmonary TB only	New case	.0274	.	Yes	No	13900	10	249	Completed treatment	6.00	No	No
283	2008	Male	46	University	Married	Urban	Smear-negative Pulmonary TB only	New case	.2822	.	No	No	7800	8	540	Completed treatment	6.00	No	No
284	2009	Female	23	University	Single	Urban	Smear-negative Pulmonary TB only	New case	.0000	39	No	No	2110	9	12	Completed treatment	6.00	Yes	Yes
285	2009	Female	45	Primary	Single	Urban	Smear-negative Pulmonary TB only	New case	.0027	59	No	Yes	2300	8	19	Completed treatment	6.00	Yes	Yes
286	2009	Male	35	No formal	Single	Urban	Smear-negative Pulmonary TB only	New case	.0000	55	No	Yes	7900	7	68	Completed treatment	6.00	Yes	Yes
287	2009	Female	70	No formal	Widow	Urban	Smear-negative Pulmonary TB only	New case	.0795	.	Yes	No	10200	9	128	Completed treatment	6.00	No	No
288	2009	Female	22	Secondary	Single	Urban	Smear-negative Pulmonary TB only	New case	.0740	65	No	No	4900	9	213	Completed treatment	6.00	No	No
289	2010	Female	60	Secondary	Single	Urban	Smear-negative Pulmonary TB only	New case	1.7178	64	No	No	1200	7	13	Completed treatment	6.00	Yes	Yes
290	2010	Male	52	Secondary	Divorced	Urban	Smear-negative Pulmonary TB only	New case	8.0082	67	No	No	7800	10	38	Completed treatment	6.00	Yes	Yes
291	2010	Female	56	Secondary	Widow	Urban	Smear-negative Pulmonary TB only	New case	.0192	60	No	No	3800	10	105	Completed treatment	6.00	Yes	Yes
292	2010	Female	21	Secondary	Single	Urban	Smear-negative Pulmonary TB only	New case	.2932	60	No	No	4800	14	145	Completed treatment	6.00	Yes	Yes
293	2010	Female	33	Secondary	Single	Urban	Smear-negative Pulmonary TB only	Retreatment case	1.7315	49	No	No	2500	12	174	Completed treatment	6.00	Yes	Yes
294	2011	Female	26	Secondary	Single	Urban	Smear-negative Pulmonary TB only	New case	.0000	47	No	No	1400	3	.	Completed treatment	6.00	Yes	No
295	2011	Male	40	University	Single	Urban	Smear-negative Pulmonary TB only	New case	.2877	.	No	Yes	2800	8	4	Completed treatment	6.00	Yes	Yes
296	2011	Female	33	University	Single	Urban	Smear-negative Pulmonary TB only	New case	.0932	34	No	No	2700	5	11	Completed treatment	6.00	Yes	Yes
297	2011	Female	33	Primary	Single	Urban	Smear-negative Pulmonary TB only	New case	.9342	58	No	No	10700	10	43	Completed treatment	6.00	Yes	Yes
298	2011	Male	25	Secondary	Single	Urban	Smear-negative Pulmonary TB only	New case	.0767	53	No	No	1600	7	96	Completed treatment	6.00	No	No
299	2011	Male	33	University	Married	Urban	Smear-negative Pulmonary TB only	New case	.6493	64	No	No	5400	15	111	Completed treatment	6.00	Yes	No
300	2011	Female	37	Secondary	Married	Urban	Smear-negative Pulmonary TB only	New case	.4712	.	No	No	4290	9	128	Completed treatment	6.00	Yes	Yes
301	2012	Male	54	Primary	Widow	Urban	Smear-negative Pulmonary TB only	Retreatment case	11.3863	58	No	No	12500	10	12	Completed treatment	6.00	Yes	Yes
302	2012	Male	31	Primary	Single	Rural	Smear-negative Pulmonary TB only	New case	.2411	54	No	No	9320	11	34	Completed treatment	6.00	Yes	Yes
303	2012	Male	48	University	Divorced	Urban	Smear-negative Pulmonary TB only	New case	.0356	80	No	No	9200	6	51	Completed treatment	6.00	Yes	Yes
304	2013	Female	47	No formal	Widow	Urban	Smear-negative Pulmonary TB only	New case	.0356	55	No	No	1100	6	.	Completed treatment	6.00	Yes	No
305	2013	Male	31	No formal	Single	Urban	Smear-negative Pulmonary TB only	New case	.0329	54	No	No	9500	11	12	Completed treatment	6.00	Yes	Yes
306	2013	Male	56	Secondary	Married	Urban	Smear-negative Pulmonary TB only	New case	1.1151	42	No	No	5130	8	57	Completed treatment	6.00	No	No
307	2006	Female	35	No formal	Married	Urban	Smear-Positive Pulmonary TB only	Retreatment case	3.2438	58	No	No	2640	5	14	Completed treatment	6.00	Yes	Yes
308	2006	Male	43	University	Married	Urban	Smear-Positive Pulmonary TB only	New case	.2877	78	Yes	No	13700	8	16	Completed treatment	6.00	Yes	Yes
309	2006	Female	33	No formal	Single	Urban	Smear-Positive Pulmonary TB only	New case	.1507	.	No	No	5200	10	50	Completed treatment	6.00	Yes	Yes
310	2006	Female	43	Secondary	Married	Urban	Smear-Positive Pulmonary TB only	New case	.0192	55	No	No	6800	11	255	Completed treatment	6.00	Yes	No
311	2007	Female	56	Secondary	Widow	Urban	Smear-Positive Pulmonary TB only	New case	.8712	50	No	No	3520	10	128	Completed treatment	6.00	No	Yes
312	2007	Female	30	Secondary	Single	Urban	Smear-Positive Pulmonary TB only	New case	.2329	48	No	No	4600	5	142	Completed treatment	6.00	Yes	No
313	2007	Female	23	University	Single	Urban	Smear-Positive Pulmonary TB only	New case	.0110	41	No	No	4200	10	321	Completed treatment	6.00	Yes	No
314	2008	Male	75	University	Married	Urban	Smear-Positive Pulmonary TB only	New case	2.0027	.	No	No	.	.	.	Completed treatment	6.00	No	No
315	2008	Male	40	University	Single	Urban	Smear-Positive Pulmonary TB only	New case	.0000	.	No	No	4200	11	.	Completed treatment	6.00	Yes	No
316	2008	Female	31	Secondary	Single	Urban	Smear-Positive Pulmonary TB only	New case	3.1699	72	No	No	5100	7	.	Completed treatment	6.00	No	No
317	2008	Male	46	University	Married	Urban	Smear-Positive Pulmonary TB only	Retreatment case	.0055	54	No	No	14100	8	74	Completed treatment	6.00	Yes	Yes
318	2008	Female	32	Secondary	Single	Urban	Smear-Positive Pulmonary TB only	New case	.1945	44	No	No	7200	9	102	Completed treatment	6.00	Yes	Yes
319	2008	Female	28	Secondary	Married	Urban	Smear-Positive Pulmonary TB only	New case	2.4521	32	Yes	No	14400	8	116	Completed treatment	6.00	Yes	Yes
320	2009	Male	53	University	Married	Urban	Smear-Positive Pulmonary TB only	New case	.8356	.	No	No	.	.	.	Completed treatment	6.00	Yes	Yes
321	2009	Female	30	University	Single	Urban	Smear-Positive Pulmonary TB only	New case	.0000	45	No	No	12700	6	71	Completed treatment	6.00	Yes	Yes
322	2010	Female	31	University	Married	Urban	Smear-Positive Pulmonary TB only	New case	.0301	.	No	No	4500	10	142	Completed treatment	6.00	No	Yes
323	2010	Female	24	Secondary	Single	Urban	Smear-Positive Pulmonary TB only	New case	.0027	57	No	No	6700	11	154	Completed treatment	6.00	No	Yes
324	2010	Female	38	Secondary	Married	Urban	Smear-Positive Pulmonary TB only	New case	.0027	59	No	No	3400	7	234	Completed treatment	6.00	Yes	Yes
325	2011	Female	21	Primary	Single	Urban	Smear-Positive Pulmonary TB only	New case	.0000	30	No	No	.	.	.	Completed treatment	6.00	Yes	No
326	2011	Male	34	Secondary	Married	Urban	Smear-Positive Pulmonary TB only	New case	.6055	53	No	No	4900	7	.	Completed treatment	6.00	Yes	Yes
327	2011	Male	41	University	Married	Urban	Smear-Positive Pulmonary TB only	New case	.0027	56	No	No	2650	12	25	Completed treatment	6.00	Yes	Yes
328	2011	Female	34	University	Widow	Urban	Smear-Positive Pulmonary TB only	New case	4.1589	48	Yes	No	3260	10	30	Completed treatment	6.00	Yes	Yes
329	2011	Male	44	Secondary	Married	Urban	Smear-Positive Pulmonary TB only	New case	.0932	58	No	No	2400	10	70	Completed treatment	6.00	No	Yes
330	2011	Female	26	University	Single	Urban	Smear-Positive Pulmonary TB only	New case	.1233	74	No	No	7700	4	117	Completed treatment	6.00	Yes	No
331	2011	Male	36	University	Married	Urban	Smear-Positive Pulmonary TB only	New case	.0219	65	No	No	5500	8	156	Completed treatment	6.00	Yes	Yes
332	2012	Female	50	University	Married	Urban	Smear-Positive Pulmonary TB only	New case	.0000	42	Yes	No	4570	9	187	Completed treatment	6.00	Yes	Yes
333	2013	Female	35	Secondary	Married	Urban	Smear-Positive Pulmonary TB only	New case	.3288	67	No	No	2700	8	10	Completed treatment	6.00	Yes	Yes

334	2013	Female	31	University	Single	Urban	Smear-Positive Pulmonary TB only	New case	.3781	48	No	No	5400	11	54	Completed treatment	6.00	Yes	Yes
335	2013	Female	32	No formal	Single	Urban	Smear-Positive Pulmonary TB only	New case	3.0301	58	No	Yes	2700	6	118	Completed treatment	6.00	Yes	Yes
336	2013	Female	30	No formal	Single	Urban	Smear-Positive Pulmonary TB only	New case	.7890	45	No	No	7600	8	142	Completed treatment	6.00	Yes	No
337	2013	Male	51	Primary	Married	Rural	Smear-Positive Pulmonary TB only	New case	.0000	62	No	No	2300	7	184	Completed treatment	6.00	No	No
N	337	337	337	337	337	337	337	337	337	301	337	337	319	319	309	337	337	337	337

TB: tuberculosis

Mixed form: Extra-pulmonary TB + Pulmonary TB
